# Human MCTS1-dependent translation of JAK2 is essential for IFN-γ immunity to mycobacteria

**DOI:** 10.1016/j.cell.2023.09.024

**Published:** 2023-10-23

**Authors:** Jonathan Bohlen, Qinhua Zhou, Quentin Philippot, Masato Ogishi, Darawan Rinchai, Tea Nieminen, Simin Seyedpour, Nima Parvaneh, Nima Rezaei, Niloufar Yazdanpanah, Mana Momenilandi, Clément Conil, Anna-Lena Neehus, Carltin Schmidt, Carlos Andres Arango Franco, Tom Le Voyer, Taushif Khan, Rui Yang, Julia Puchan, Lucia Erazo, Mykola Roiuk, Taja Vatovec, Zarah Janda, Ivan Bagaric, Marie Materna, Adrian Gervais, Hailun Li, Jérémie Rosain, Jessica Peel, Yoann Seeleuthner, Ji Eun Han, Anne-Sophie L’Honneur, Marcela Moncada-Vélez, Marta Martin-Fernandez, Michael Espino Horesh, Tatiana Kochetkov, Monika Schmidt, Mohammed A. AlShehri, Eeva Salo, Saxen Harri, Gehad ElGhazali, Ahmad Yatim, Camille Soudée, Federica Sallusto, Armin Ensser, Nico Marr, Peng Zhang, Dusan Bogunovic, Aurélie Cobat, Mohammad Shahrooei, Vivien Béziat, Laurent Abel, Xiaochuan Wang, Stéphanie Boisson-Dupuis, Aurelio A. Teleman, Jacinta Bustamante, Qian Zhang, Jean-Laurent Casanova

**Affiliations:** 1 Laboratory of Human Genetics of Infectious Diseases, Necker Hospital for Sick Children, 75015 Paris, France; 2 Paris Cité University, Imagine Institute, 75015 Paris, France; 3 German Cancer Research Center (DKFZ), 69120 Heidelberg, Germany; 4 Heidelberg University, 69120 Heidelberg, Germany; 5 St. Giles Laboratory of Human Genetics of Infectious Diseases, The Rockefeller University, 10065 NY, USA; 6 Children’s Hospital of Fudan University, 201102 Shanghai, China; 7 New Children’s Hospital, 00290 Helsinki, Finland; 8 Research Center for Immunodeficiencies, University of Medical Sciences, P94V+8MF Tehran, Iran; 9 Nanomedicine Research Association (NRA), P94V+8MF Tehran, Iran; 10 Department of Pediatrics, Tehran University of Medical Sciences, P94V+8MF Tehran, Iran; 11 Children’s Medical Center, P94V+8MF Tehran, Iran; 12 Network of Immunity in Infection, Malignancy and Autoimmunity (NIIMA), P94V+8MF Tehran, Iran; 13 Faculty of Medicine, Heinrich Heine University Düsseldorf, 40225 Düsseldorf, Germany; 14 College of Health and Life Sciences, Hamad Bin Khalifa University, 8C8M+6Q Doha, Qatar; 15 Department of Immunology, Sidra Medicine, 8C8M+6Q Doha, Qatar; 16 The Jackson Laboratory, Farmington, CT-USA; 17 Institute of Microbiology, ETH Zürich, 8049 Zürich, Switzerland; 18 Department of Virology, Cochin Hospital, 75015 Paris, France, EU; 19 Center for Inborn Errors of Immunity, Icahn School of Medicine at Mount Sinai, 10029 NY, USA; 20 Precision Immunology Institute, Icahn School, 10029New York, NY, USA; 21 Mindich Child Health and Development Institute, Icahn School, 10029 New York, NY, USA; 22 Department of Pediatrics, Icahn School, 10029 New York, NY, USA; 23 Department of Microbiology, Icahn School, 10029 New York, NY, USA; 24 Institute of Clinical and Molecular Virology, Universitätsklinikum Erlangen, 91054 Erlangen, Germany; 25 King Fahad Medical City, Children’s Specialized Hospital, 12231 Riyadh, Saudi-Arabia; 26 Sheikh Khalifa Medical City, Union71-Purehealth, United Arab Emirates University, Al Ain, United Arab Emirates; 27 Institute for Research in Biomedicine, Università della Svizzera Italiana, 6500 Bellinzona, Switzerland; 28 Clinical and Diagnostic Immunology, KU Leuven, 3000 Leuven, Belgium; 29 Dr. Shahrooei Laboratory, 22 Bahman St., Ashrafi Esfahani Blvd, Tehran, Iran; 30 Study Center for Primary Immunodeficiencies, AP-HP, 75015 Paris, France; 31 Howard Hughes Medical Institute, 10032 New York, NY, USA; 32 Department of Pediatrics, Necker Hospital, 75015 Paris, France, EU

**Keywords:** MCTS1, translation reinitiation, JAK2, IL-23, mycobacterium, MSMD, inborn error of immunity, X-linked disease

## Abstract

Human inherited disorders of IFN-γ immunity underlie severe mycobacterial diseases. We report X-linked recessive MCTS1 deficiency in men with mycobacterial disease from kindreds of different ancestries (from China, Finland, Iran, and Saudi Arabia). Complete deficiency of this translation re-initiation factor impairs the translation of a subset of proteins, including the kinase JAK2 in all cell types tested, including T lymphocytes and phagocytes. JAK2 expression is sufficiently low to impair cellular responses to IL-23 and partially IL-12, but not other JAK2-dependent cytokines. Defective responses to IL-23 preferentially impair the production of IFN-γ by innate-like adaptive MAIT and γδ T lymphocytes upon mycobacterial challenge. Surprisingly, the lack of MCTS1-dependent translation re-initiation and ribosome recycling seems to be otherwise physiologically redundant in these patients. These findings suggest that X-linked recessive human MCTS1 deficiency underlies isolated mycobacterial disease by impairing JAK2 translation in innate-like adaptive T lymphocytes, thereby impairing the IL-23-dependent induction of IFN-γ.

## One-Sentence Summary

X-linked recessive human MCTS1 deficiency underlies mycobacterial disease by impairing JAK2 translation in innate-like T lymphocytes, thereby decreasing the IL-23-dependent production of IFN-γ by these cells upon mycobacterial challenge.

## Graphical Abstract

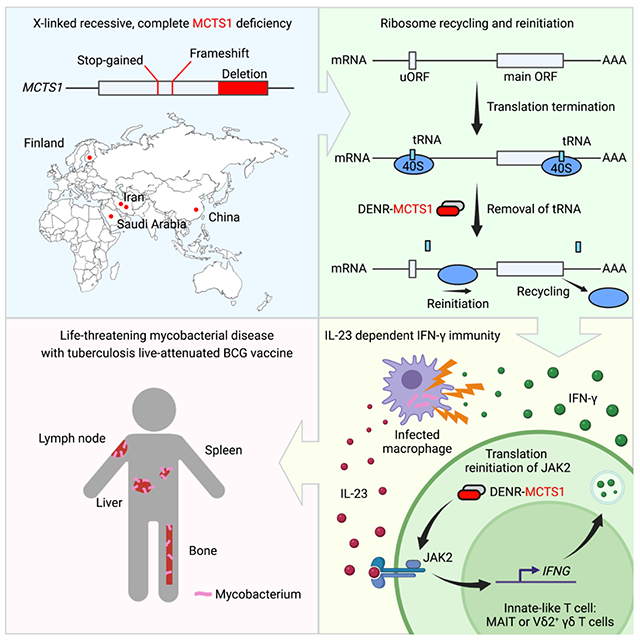

## Introduction

The clinical outcome of any infection varies considerably between individuals, ranging from silent infection in most, to lethal disease in a few^1,2^. Tuberculosis (TB), caused by *Mycobacterium tuberculosis*, has been a deadly infectious disease, with at least one billion deaths in the last 2,000 years^3^. However, infection with *M. tuberculosis* is silent or benign in >90% of infected individuals^3^. Forward genetics studies of rare patients with clinical disease due to the weakly virulent Bacillus Calmette–Guérin (BCG) live-attenuated vaccine against TB, or atypical environmental mycobacteria (EM) led to the discovery of human genetic and immunological determinants of TB^4–8^. Severe disease due to BCG or EM^9^ in otherwise healthy individuals, particularly those without HIV infection or immunosuppression, is referred to as Mendelian susceptibility to mycobacterial disease (MSMD) because of the frequent occurrence of multiplex families and parental consanguinity^10^. MSMD is relatively rare, occurring in ~1 in 50,000 individuals^11,12^, and is typically ‘isolated’ (~75% of cases) or, rarely, ‘syndromic’ (~25%)^12–14^. Patients with isolated MSMD are otherwise healthy and normally resistant to most common microbes, except, occasionally, other intramacrophagic pathogens, including some bacteria (e.g. *Salmonella*), fungi (e.g. *Coccidioidomyces*), and parasites (e.g. *Leishmania*)^12^.

Over the last 25 years, the discovery of inborn errors of interferon-gamma (IFN-γ) immunity has defined the root cause and immunological mechanism of MSMD and TB^11,14,16^. With one possible exception (ZNFX1 deficiency), all genetic defects underlying both isolated and syndromic MSMD perturb IFN-γ immunity (germline mutations of genes encoding IFN-γR1, IFN-γR2, IFN-γ, STAT1, IRF1, JAK1, IL-12Rß1, IL-12Rß2, IL-23R, IL-12p40, TYK2, SPPL2A, IRF8, NEMO, CYBB, ISG15, USP18, RORγT, and T-bet)^4–8,15,17–37^. Many genetic defects directly impair the production of IFN-γ, or the response to this cytokine (IFN-γR1, IFN-γR2, JAK1, STAT1, IRF1), whereas others affect the production of, or response to the IFN-γ-inducing cytokines IL-12, IL-23, and ISG15 (IL-12Rß1, IL-12Rß2, IL-23R, IL-12p40, TYK2, ISG15). Deficiencies of the IL-12-specific IL-12Rß2^30^ and IL-23-specific IL-23R^30,41,42^ also underlie MSMD, suggesting that neither of these two IFN-γ-inducing cytokines is dispensable for antimycobacterial immunity.

Human genetic studies of MSMD have, thus, revealed that IL-12- and IL-23-dependent IFN-γ immunity is essential for host defense against weakly virulent mycobacteria. Upon infection with mycobacteria^43^, macrophages produce IL-12 and IL-23, which stimulate natural killer (NK) and T lymphocytes to produce IFN-γ. Several lymphocyte subsets have been identified as critical producers of IFN-γ and mediators of antimycobacterial immunity: patients with autosomal recessive (AR) SPPL2A or RORγT deficiency, or with autosomal dominant (AD) IRF8 deficiency suffer from MSMD due to a lack of T-helper (T_H_) 1* cells^27,31^, whereas AR T-bet deficiency impairs the development and IFN-γ production of NK cells, innate-like adaptive lymphocyte subsets (mucosal-associated invariant T cells [MAIT], Vδ2^+^ γδ T, and invariant natural killer [iNKT] cells), and T_H_1 cells^34^. In IL-12Rß1-, IL-12Rß2-, and IL-23R-deficient patients, these subsets (NK, MAIT, Vδ2^+^ γδ T, iNKT, T_H_1 and T_H_1*) also fail to produce IFN-γ in response to IL-12 and/or IL-23^30,42^. IFN-γ can stimulate most cell types, but is crucial for the killing of mycobacteria within phagocytes^43,44^, and it also boosts the production of IL-12 and IL-23 by these cells, forming a positive feedback loop^45–48^.

Molecular genetics studies of MSMD have revealed the mechanisms of antimycobacterial immunity in humans *in natura*^9,49,50^, demonstrating that IFN-γ acts as a key antimycobacterial cytokine rather than an antiviral interferon. Severe viral diseases are rare in patients with MSMD, occurring mostly in patients with syndromic MSMD who also have impaired IFN-α/β immunity (mutations of *JAK1, STAT1*, *TYK2*)^21,22,28,52^. Moreover, the study of MSMD has led to the discovery of both rare and common determinants of clinical TB, which is caused by *M. tuberculosis*, a bacterium 1,000 times more virulent than those implicated in MSMD^2^. Most genetic etiologies of MSMD have incomplete penetrance for MSMD and can manifest as genetic etiologies of TB^2,16,35^. Moreover, homozygosity for the common P1104A allele of TYK2 selectively impairs the IL-23-dependent induction of IFN-γ and accounts for about 1% of cases of TB in patients of European descent^2,18,54^. The study of MSMD has, therefore, had both biological and medical implications. However, a clear genetic etiology has yet to be found for about half the MSMD patients identified to date, implying that our understanding of antimycobacterial immunity is incomplete. In this context, we searched for new genetic causes and immunological mechanisms of MSMD.

## Results

### Hemizygosity for pLOF variants of MCTS1 in five unrelated men with MSMD

**Figure 1:**
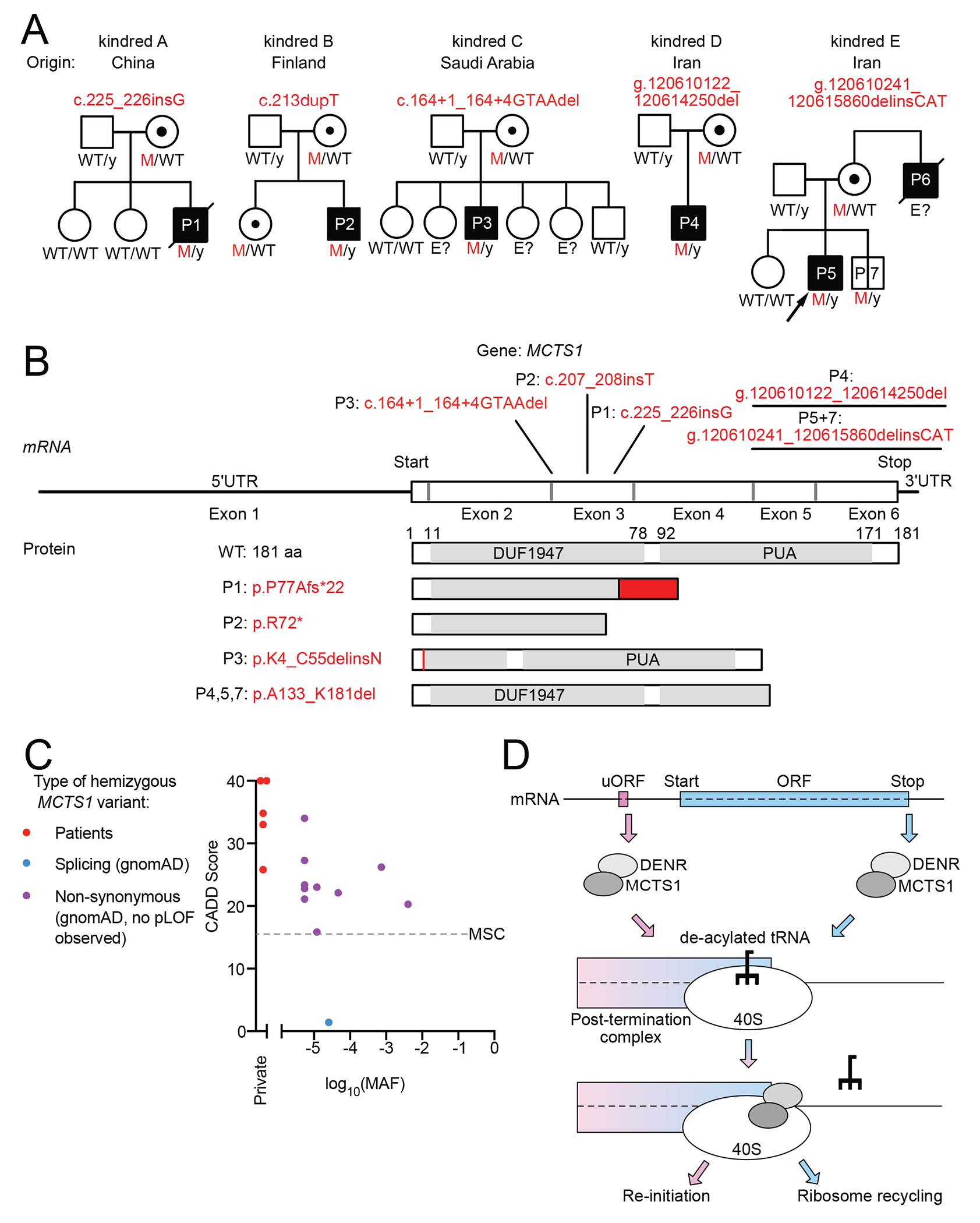
Hemizygous *MCTS1* mutations in patients with MSMD from five unrelated families. **(A)** Family pedigrees with allele segregation. The patients in black suffer from MSMD and are hemizygous for the indicated *MCTS1* alleles. The arrow indicates the proband. Other symbols; asymptomatic: black vertical line, heterozygous: blackdots, unknown genotype: “E?”, wild-type: WT. **(B)** Schematic representation of the MCTS1 mRNA (top) and protein (middle) structure. (Bottom) Predicted protein products of the *MCTS1* variants found in MSMD patients. **(C)** CADD MAF plot of all the nonsynonymous, hemizygous *MCTS1* variants of the gnomAD database (in black, splicing in blue), and the five *MCTS1* pLOF variants of patients with MSMD (in red). **(D)** Schematic representation of the known molecular function of MCTS1 and its binding partner, DENR.

We hypothesized that MSMD might be due to the X-linked recessive (XR) inheritance of a single genetic defect in at least some patients in a cohort of patients with unexplained MSMD, as men outnumber women in this cohort (422 men, 359 women). We used whole-exome sequencing (WES) to search for rare predicted loss-of-function (pLOF, meaning stop-gain, frameshift, essential splice-site and large insertion/deletion) variants present on the X chromosome. We identified five independent, private (detected only in these kindreds) pLOF variants of the *MCTS1* gene in male patients from five kindreds (Figure 1A–B). Sanger sequencing confirmed the presence and familial segregation of these variants, consistent with the XR inheritance of MSMD (Figure 1A, Figure S1A–E). The enrichment of these *MCTS1* pLOF variants in our male MSMD cohort without genetic diagnosis (*n* = 422, 5 pLOF variants) relative to a cohort comprising both male patients without mycobacterial infections and healthy male individuals (*n* = 1,932, 0 pLOF variants) was highly significant (*p* = 0.00013). The five patients come from unrelated, non-consanguineous families (Figure 1A, Case reports, Table S1). The patients are Chinese (P1), Finnish (P2), Saudi Arabian (P3), and Iranian (P4, P5) and are of East Asian, Finnish, Semitic, and Indo-European ancestries, respectively (Figure S1F). All patients were vaccinated with BCG within a day of birth (Table S1). The patients developed life-threatening BCG disease three months to one year after vaccination. Disease was multifocal or disseminated in several cases (P1-P3, P5), and involved osteomyelitis in P1 and P3. P1 died from BCG disease at the age of two years. The maternal uncle of P5 (P6) died at the age of two years from a condition consistent with MSMD (no genetic material available). P2, P3, P4, and P5 survived BCG infection and are now healthy at the ages of 18, 12, 6 and 3 years, respectively. There was no history of any other unusually severe infectious or non-infectious disease in these patients, suggesting a diagnosis of isolated MSMD. The brother of P5 (P7) was born in 2021, and was diagnosed as hemizygous for the familial *MCTS1* variant six days after birth; BCG vaccination was, thus, contraindicated, and he remains asymptomatic to date. Detailed clinical reports are provided in the methods section. These cases highlight the potential role of *MCTS1* variants in isolated MSMD.

### pLOF variants of MCTS1 and DENR are absent from the general population

All five pLOF variants of the *MCTS1* gene identified in the five index patients are private, with combined annotation-dependent depletion (CADD) scores well above the mutation significance cutoff (MSC) for *MCTS1*^55,56^ (Figure 1C), further suggesting that they are deleterious. No pLOF variant of *MCTS1* in the hemizygous, homozygous, or heterozygous state was reported in the gnomAD dataset (v2, 125,748 whole-exome and 15,708 whole-genome sequences). Ten missense variants of *MCTS1 (ENST00000371317*) in the hemizygous state are reported in gnomAD (Figure 1C). A search of our in-house WES and WGS (whole-genome sequencing) datasets from more than 15,000 individuals with various infectious diseases revealed no additional hemizygous pLOF variants of *MCTS1*. The probability of *MCTS1* being loss-of-function-intolerant (pLI) was found to be 0.87, indicating that truncating variants are not tolerated in the general population. Density-regulated re-initiation and release factor (DENR) is the constitutive binding partner of MCTS1, and these two proteins are interdependent for ribosome recycling and re-initiation activity (Figure 1D)^58,59^. DENR knockout (KO) and MCTS1 knockdown (KD) are lethal during the development of *Drosophila melanogaster* because they impair histoblast proliferation^58^. No studies of DENR^KO^ mice or MCTS1^KO^ animal models have yet been published, but International Mouse Phenotyping Consortium (IMPC) data suggest that DENR^KO^ is embryonically lethal in mice, whereas MCTS1^KO^ mice are viable and have no obvious phenotype^60^ (the DENR and MCTS1 KO phenotypes are summarized in Table S2). DENR pLOF variants are very rare in gnomAD, with a cumulative allele frequency of 5.5*10^−5^ and no homozygous carriers detected. We found no individuals heterozygous or homozygous for pLOF variants of *DENR* in our own cohort of patients with various infectious diseases, including MSMD. Collectively, these data suggest that the hemizygous pLOF variants of *MCTS1* identified in these patients from five unrelated kindreds probably underlie their MSMD.

### The patients’ variants are loss-of-expression (LOE)

**Figure 2:**
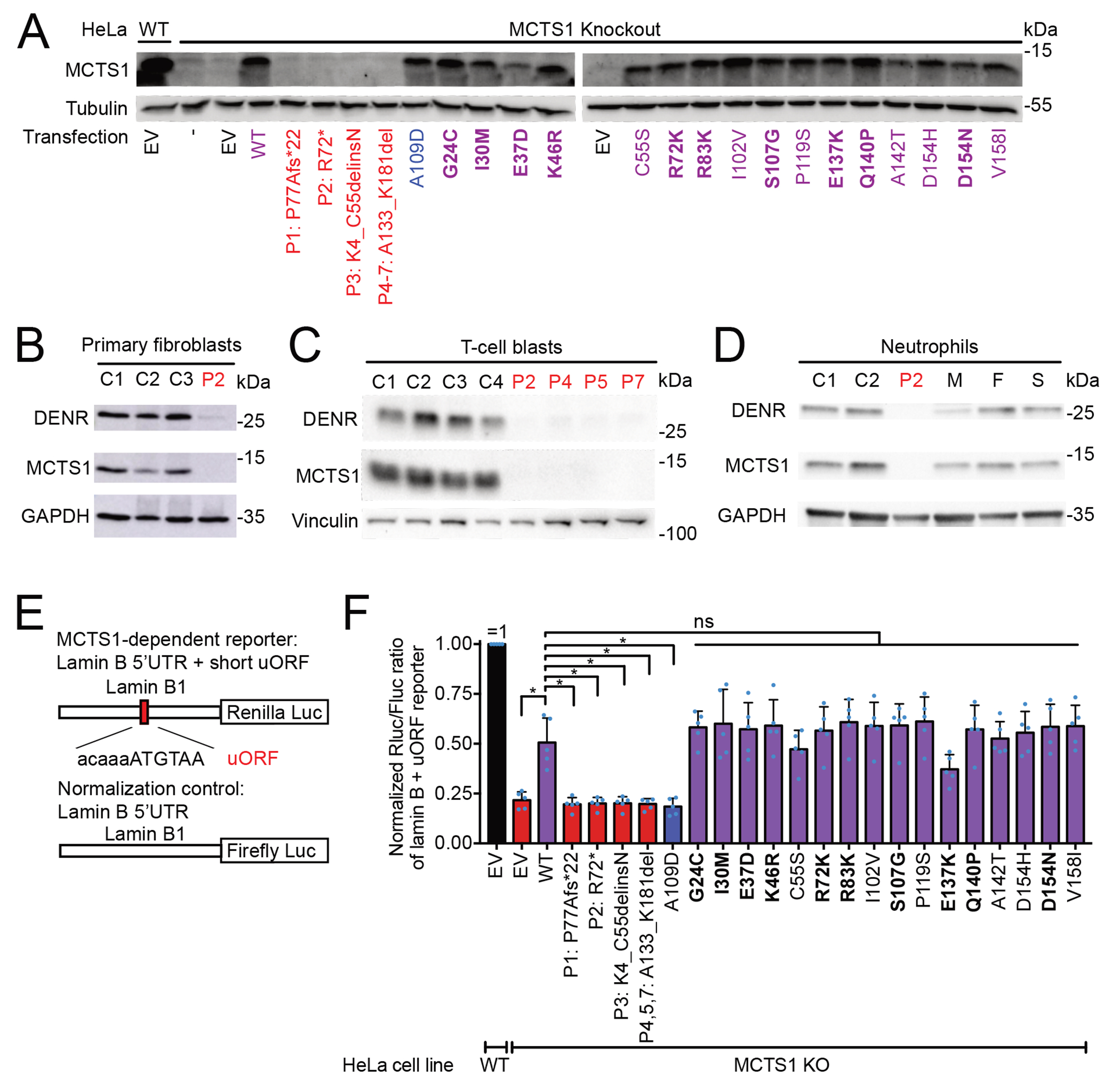
The *MCTS1* mutations found in MSMD patients are loss-of-expression and loss-of-function. **(A)** Western blot of wild-type (WT) and MCTS1^KO^ HeLa cells transiently transfected with MCTS1 variants; Red: patients; blue: synthetic LOF variant; purple: gnomAD missense variants; EV: empty vector. Bold variants: hemizygous in gnomAD. Representative data from three independent experiments are shown. **(B-D)** Levels of MCTS1 protein in **(B)** primary fibroblasts from P2 and healthy controls, **(C)** T-cell blasts from P2, P4, P5 and P7 and healthy WT controls, and **(D)** neutrophils of P2, his relatives (M = mother, F= father, S = sister) and two healthy WT controls (C1, C2) as determined by western blotting. **(E)** Schematic representation of the reporter constructs used for the MCTS1 activity assay in **(F)** Activity of the MCTS1 variants used for the transient transfection of MCTS1^KO^ HeLa cells, as assessed by the luciferase translation reinitiation assay. Bars: mean and SD of five biological replicates. Statistical significance was assessed in unpaired Welch’s *t*-tests corrected for multiple testing. *p<0.05

*MCTS1* (ENSG00000232119, ENST00000371317.10) encodes malignant T cell-amplified sequence 1, a 181-amino acid polypeptide composed of a domain of unknown function (DUF1947) and an RNA-binding domain (PUA) (Figure 1B). P1’s variant (c.225_226insG) causes a frameshift resulting in a premature stop codon (p.P77Afs*22, Figure 1B). P2’s variant (c.213dupT) creates a premature stop codon (p.R72*). The variant found in P3 (c.164+1_164+4delGTAA) is predicted to disrupt the consensus sequence for the splicing donor site of *MCTS1* intron 2. Indeed, exon trapping in COS-7 cells and the sequencing of whole-blood RNA from P3 and his parents confirmed a severe impairment of splicing at this site, with no production of the canonical transcript in COS-7 cells (Figure S2A) and less than 3% of reads supporting normal splicing in P3’s blood (Figure S2B, C). In more than 90% of splicing events, exon 2 is skipped, resulting in a truncated MCTS1 protein lacking the 51 amino acids encoded by exon 2 (p.K4_C55delinsN). The residual splicing events skip both exon 2 and exon 3 or retain intron 2, leading, in both cases, to a frameshift and the gain of a premature stop codon. Finally, the *MCTS1* alleles of P4, P5 and P7 present two different large deletions (4,128 and 5,619 nucleotides) affecting the two exons at the 3’-end of *MCTS1* (P4: g.120610122_120614250del; P5 and P7:g.120610241_120615860delinsCAT) (Figure S2D). Both lead to predicted truncation of the MCTS1 protein (p.A133_K181del). We assessed protein production for the patients’ *MCTS1* variants. We transiently transfected MCTS1^KO^ HeLa cells^59^ with wildtype (WT) or variant *MCTS1* cDNA constructs and assessed MCTS1 protein levels by western blotting (Figure 2A). None of the five *MCTS1* alleles found in MSMD patients drove the expression of a full-length or truncated MCTS1 proteins, whereas all the missense *MCTS1* variants found in gnomAD drove MCTS1 expression. We also observed a lack of MCTS1 production and low levels of DENR protein in the primary fibroblasts of P2 (Figure 2B), T-cell blasts (T-blasts) from P2, P4, P5, and P7 (Figure 2C), and primary neutrophils from P2 (Figure 2D). The heterozygous female carriers tested — the sister and mother of P2 — and the wild-type father of P2, had normal levels of MCTS1 protein in primary neutrophils (Figure 2D). Thus, none of the *MCTS1* variants encodes a detectable MCTS1 protein when overexpressed in recipient cells or in primary hemizygous cells.

### The patients’ variants are loss-of-function for translation re-initiation

*MCTS1* and *DENR* are ubiquitously expressed in all cell types and tissues in humans^61^. Following the translation of an open-reading frame (ORF) by a ribosome, the DENR-MCTS1 complex removes the tRNA from the 40S ribosome on the stop codon, as a part of ribosome recycling (Figure 1D). In eukaryotic cells, there are at least two types of ORFs: classical, protein-coding ORFs (main ORFs, mORF) and upstream ORFs (uORF), which are small coding sequences in the 5’UTR of mRNAs. These uORFs occur in about half of all mammalian mRNAs and downregulate the translation of mORFs^60^. We hypothesized that XR MCTS1 deficiency might disturb ribosome recycling at two types of stop codons: classical mORF and 5’UTR uORF stop codons. We first assessed MCTS1 function on uORF stop codons, for which MCTS1 promotes the re-initiation of translation^58,59^. In the absence of MCTS1 or DENR, 40S ribosomes stall on the uORF stop codon, because the deacylated tRNA cannot be removed^59^. This has a roadblock-like effect, leading to poor expression of the corresponding mORF of the mRNA, due to impaired translation re-initiation. We assessed the five *MCTS1* LOE mutations in a luciferase-based translation re-initiation assay (Figure 2E). This assay uses a reporter plasmid with a short uORF in its 5’UTR, rendering expression of the reporter gene dependent on the MCTS1-DENR complex^58,59^. The *MCTS1* variants found in MSMD patients were loss-of-function (LOF) for MCTS1 activity (Figure 2F), like the synthetic A109D LOF mutation used as a negative control^62^. All 16 non-synonymous variants found in the general population were tested and found to have activity levels similar to those of the (WT) MCTS1 (isomorphic variants). *MCTS2P* is an *MCTS1* paralog and pseudogene located on a different chromosome (Chr. 20). The predicted protein sequence of MCTS2P is ~95% identical to that of MCTS1 (Figure S2E). In our functional assay, MCTS2P had detectable but weak activity with the DENR-MCTS1-dependent reporter (Figure S2I). However, queries against all the RNA-sequencing datasets produced in this manuscript showed that none of the total of 4.7 billion reads mapped uniquely to *MCTS2P*, whereas 403,296 reads mapped to *MCTS1* (Figure S2F–H). Consistently, CAGE data from the FANTOM5 project suggest that the *MCTS1* promoter is much stronger (score 33.8) than the putative *MCTS2P* promoter (score 0.69)^63,64^. These data suggest that *MCTS2P* is a non-functional paralog of *MCTS1*, due to its lack of transcription. Collectively, these results suggest that the *MCTS1* variants found in the MSMD patients from the five unrelated kindreds abolish MCTS1 reinitiation activity, resulting in complete MCTS1 deficiency.

### Ribosomal recycling is impaired in the patients’ cells

**Figure 3:**
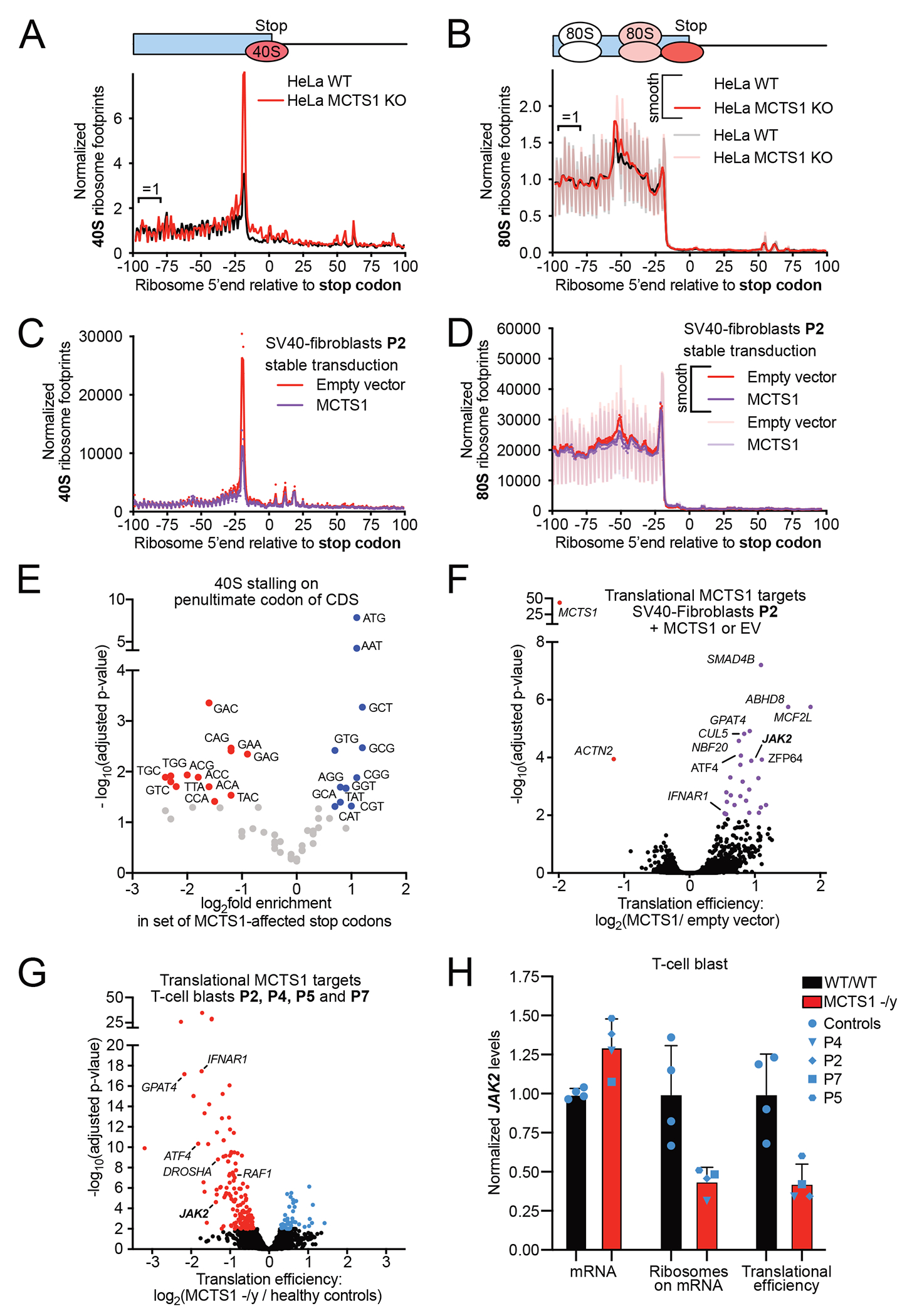
Defective ribosome recycling and translation reinitiation in the patients’ cells **(A-D)** Metagene profiles for 40S **(A, C)** and 80S **(B, D)** ribosome footprinting showing the position of the 5’ end of the ribosome footprints relative to the stop codons of all proteins (coding ORFs) in HeLa cells **(A, B)** and SV40-fibroblasts **(C, D). (A, B)** Read counts normalized against the area indicated on the plot; **(C, D)** Read counts normalized by sequencing depth. **(E)** Penultimate codon enrichments for mORFs in the top quartile for 40S accumulation (*n* = 1034) relative to all detected mORF stop codons (*n* = 4222). Significance was assessed in binomial tests, with correction for multiple testing. **(F)** Volcano plot of changes in translation efficiency from 80S footprinting data for P2 SV40-fibroblasts +/− MCTS1. The *p*-values adjusted for multiple testing in X-tail analysis are shown on the *y*-axis. **(G)** Volcano plot of changes in translation efficiency from the 80S footprinting data of T-cell blasts from P2, P4, P5, P7 relative to four healthy controls. The *p*-values adjusted for multiple testing by X-tail analysis are shown on the *y*-axis. **(H)** Levels of mRNA, ribosome footprints on mRNA and translation efficiency for the *JAK2* endogenous mRNA from **(G)**.

We hypothesized that cells lacking MCTS1 might display ribosome recycling defects. We tested this hypothesis by performing 40S and 80S ribosome footprinting (Figure S3A) on MCTS1^KO^ and WT HeLa cells (Figure S3B–D)^59,65,66^. This method reveals the position and number of 40S and 80S ribosomes on endogenous mRNAs^65,66^. We found that the frequency of stalled, post-termination 40S ribosomes was higher on mORF stop codons in MCTS1^KO^ cells (Figure 3A, indicated by arrow), as in DENR^KO^ HeLa cells, although the 40S peak for MCTS1^KO^ was smaller^59^. This finding is consistent with MCTS1 deficiency, as post-termination 40S ribosomes are the substrate of DENR-MCTS1. We observed 80S ribosome queueing in front of mORF stop codons (Figure 3B, indicated by an arrow), due to the translating 80S ribosomes running into the roadblock caused by the stalled 40S ribosome on the stop codon, as described in DENR^KO^ HeLa cells and yeast strains lacking DENR or MCTS1^59,67^ MCTS1^KO^ HeLa cells, thus, have impaired ribosome recycling. We investigated whether cells from MCTS1-deficient patients had the same molecular phenotype. We used the same 40S and 80S footprinting method on SV40-fibroblasts from P2 stably transduced with lentiviral empty vector (EV) or a lentiviral vector encoding wildtype (WT) MCTS1 (Figure S3E–G). P2’s SV40-fibroblasts had a pronounced peak of stalled post-termination 40S ribosomes on mORF (Figure 3C) and uORF stop codons (Figure S3H), which was rescued by transduction with WT *MCTS1*. The queueing 80S ribosomes in front of the stop codon were, therefore, also rescued by MCTS1 expression in P2’s cells (Figure 3D). Furthermore, cells from P2 transduced with EV presented an accumulation of 40S ribosomes on mORF (Figure 3E) and uORF (Figure S3I) stop codons in the presence of certain penultimate (position −1 relative to the stop) codons, such as ATG^Met^ and GCG^Leu^, another phenocopy of DENR^KO^ HeLa cells (Figure S3J–K). We have previously shown that this ribosome recycling defect does not negatively affect the translation of the proteins concerned^59^. The patients’ cells, thus, display impaired ribosome recycling due to their lack of MCTS1, attesting to a deficiency of the two known functions of MCTS1.

### The patients’ cells are defective for translation re-initiation

Translation may be re-initiated in almost half of all human genes^60^. The MCTS1-DENR complex is specifically required for translation re-initiation following uORFs that are either very short^58,62^ or terminate in certain penultimate codons^59^ (~200 of 8,900 detected genes^59^). We identified the genes poorly translated in MCTS1-deficient cells by comparing the translation efficiency of all detected genes (*n* = 10,505) between P2 SV40-fibroblasts transduced with EV and with WT MCTS1 (Figure 3F). Xtail^68^ analysis identified 26 genes as having significantly higher levels of translation after MCTS1 rescue in P2’s cells (Figure 3F), with translation levels decreasing for only two genes following such rescue (Table S3). *JAK2* is the only target gene directly related to IFN-γ immunity, as JAK2 is required for the IL-12- and IL-23-dependent induction of IFN-γ and the response to IFN-γ^18,70^. Another affected gene important for cytokine signaling is *IFNAR1*, encoding a chain of the IFN-α/β receptor (Figure 3F). Patients with germline mutations of *IFNAR1* and related genes suffer from viral, but not mycobacterial disease^2^. We investigated the translational targets of MCTS1 in a more relevant cell type, by performing 80S ribosome footprinting in primary T-cell blasts from four healthy male individuals and four MCTS1-deficient patients (P2, P4, P5 and P7). We again found that many more genes had lower levels of translation (143 genes) in the patients’ cells than had higher levels of translation (46 genes) (Figure 3G). We observed a substantial and significant overlap (*n* = 98, *p* < 10^−20^) between the DENR-dependent genes identified in DENR^KO^ HeLa cells (*n* = 275, p < 0.1) and the MCTS1-dependent genes in identified in MCTS1-deficient T-cell blasts (*n* = 297, *p* < 0.1) (Figure S3L, Table S4). The cells of MCTS1-deficient patients, like DENR^KO^ cells, therefore have a genome-wide translation re-initiation defect. *IFNAR1* was also affected, as in SV40-fibroblasts (Table S5). JAK2 translation levels were less than half those in wild-type cells (Figure 3G–H). We also assessed the translation efficiency of 25 genes related to IFN-γ immunity, including 16 of the 20 genes underlying MSMD endogenously expressed in these T-cell blasts; we found that only *JAK2* and *IRF8* were substantially affected in the patients’ cells (Figure S3M). *IRF8* mRNA levels are upregulated in MCTS1-deficient patients (Figure S3N), potentially compensating for the lower efficiency of translation at mRNA level, whereas *JAK2* mRNA levels were unaffected. Altogether, the patients’ cells presented defective translation re-initiation, resulting in the impaired translation of a subset of mRNAs, including that encoding JAK2.

### MCTS1-dependent translation re-initiation is required for JAK2 expression

**Figure 4:**
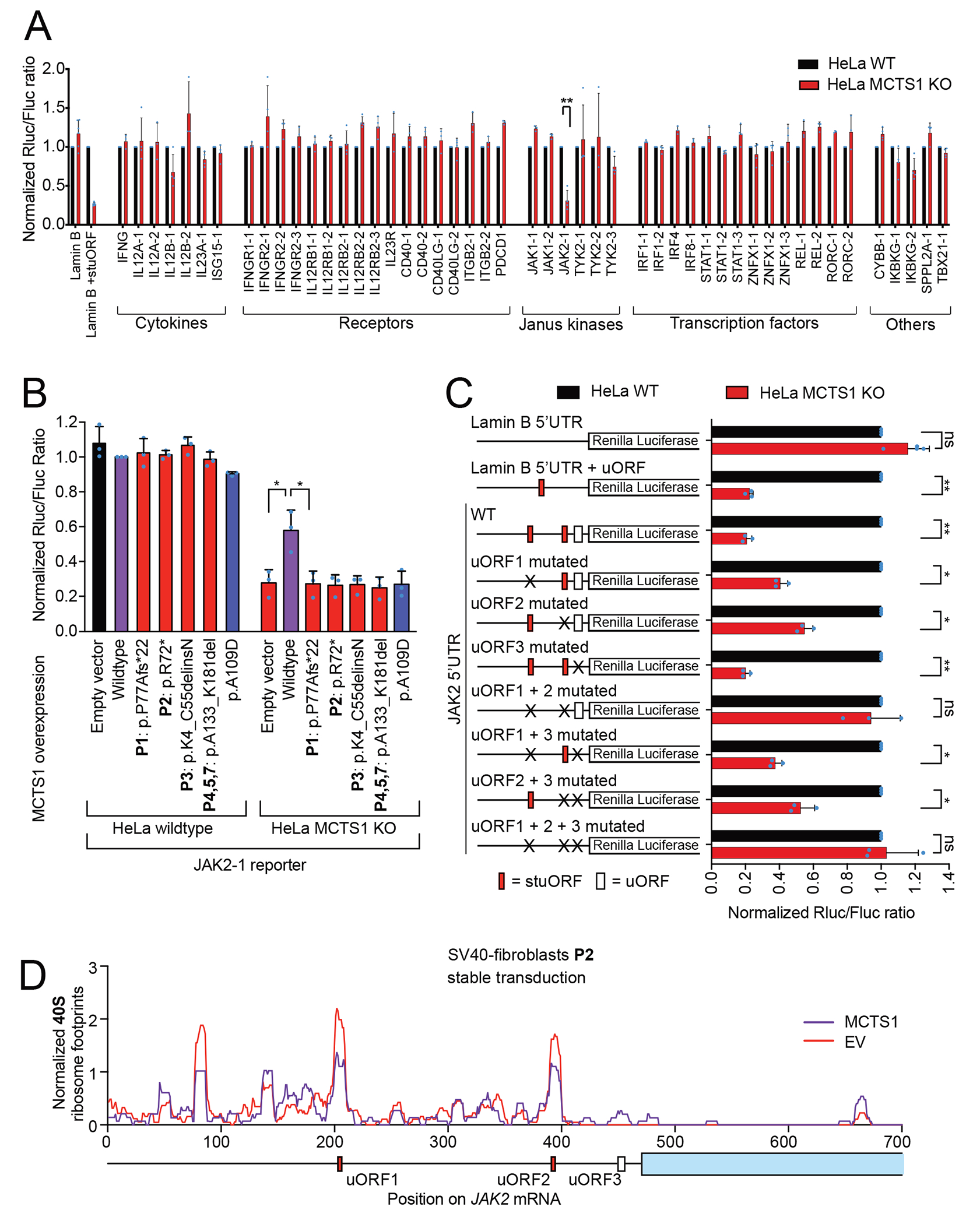
JAK2 translation depends on MCTS1-mediated reinitiation. **(A)** The 5’UTR translation reporters indicated were assessed in WT (black) and MCTS1^KO^ (red) HeLa cells. Number −1, −2 indicate distinct 5’UTR isoforms. Results are shown for 3-5 biological replicates per reporter. Statistical significance was assessed in multiple, two-tailed, unpaired *t*-tests with correction for multiple testing (**p<0.005). **(B-C)** Efficiency of JAK2 reporter translation in WT and MCTS1^KO^ cells transfected with the indicated constructs, as assessed in dual-luciferase assays. Results for three biological replicates are shown. Statistical significance was assessed in multiple, two-tailed, unpaired *t*-tests with correction for multiple testing (*p<0.05, **p<0.005). **(D)** Trace of the 40S ribosome footprints on the endogenous *JAK2* 5’UTR. Red: MCTS1-dependent stuORFs, pink: MCTS1-independent uORF, blue: JAK2 main ORF.

**Figure 5:**
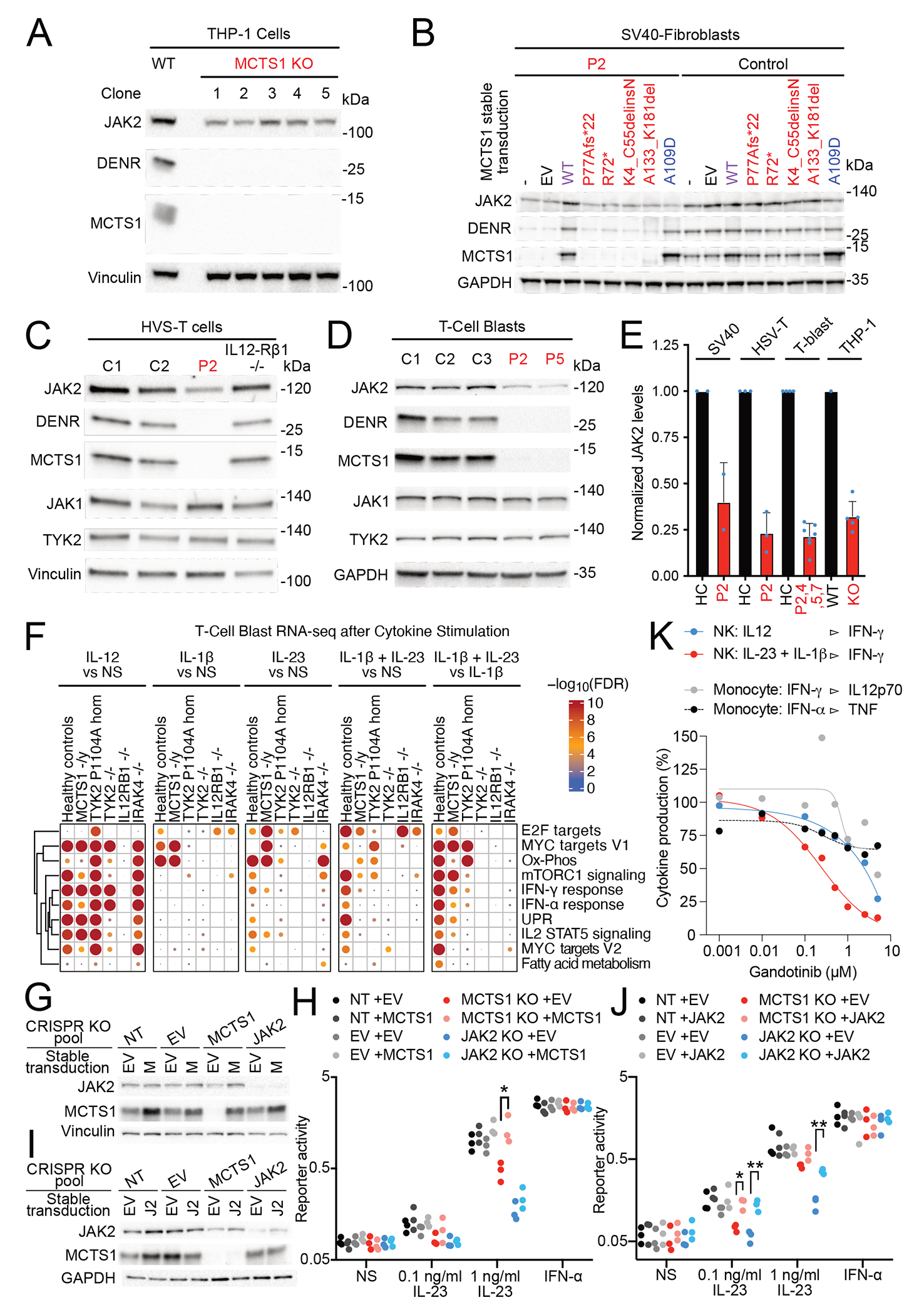
JAK2 is poorly expressed in cells derived from MCTS1-deficient patients. **(A-D)** Western blot for JAK2, MCTS1, DENR and vinculin on total lysate from **(A)** MCTS1^KO^ THP-1 clones (1 to 5) and wild-type (WT) THP-1 cells, **(B)** SV40-fibroblasts from P2 and a healthy control stably transduced with the indicated overexpression constructs, **(C)** HSV-T cells from P2, healthy controls and an IL-12Rβ1-deficient patient and **(D)** T-cell blasts from P2, P5 and healthy controls (C1, C2 and C3). The data shown are representative of three (A, C, D), or (B) two independent experiments.) **(E)** Quantification of JAK2 protein levels relative to a loading control from (A-D) **(F)** Gene set enrichment analysis of RNA-seq analysis of T-cell blasts from heathy controls (*n* = 9), MCTS1-deficient (*n* = 4), TYK2-deficient (*n* = 1), IL-12Rβ1-deficient (*n* = 1), IRAK4-deficient (*n* = 1) and TYK2 P1104A-homozygous (*n* = 2) patients were stimulated with IL-1β (24 h) and with IL-12 and IL-23 (6 h). Dot heatmaps are shown for the 10 gene sets for the healthy controls most strongly affected by stimulation with IL-23 + IL-1β relative to IL-1β alone. **(G, I)** Western blots of the indicated HEK-Blue cell lines stably transduced with the indicated lentiviral vectors. The data shown are representative of three biological replicates. **(H, J)** HEK-Blue colorimetric activity of the indicated cell lines stimulated with the indicated cytokines for 20 hours. Data are shown for three biological replicates and statistical significance was assessed in unpaired, two-tailed *t*-tests, *p<0.05, **p<0.005. **(K)** The effect of gandotinib on the response to IL-23 + IL1-β or IL-12 in NK cells and to IFN-γ or IFN-α in monocytes. Summary plot, see Figure S7O–R for details. The data shown are the means of six biological replicates performed on NK cells and monocytes from two heathy donors each.

We hypothesized that MCTS1 deficiency underlies MSMD by disrupting the translation of JAK2. We assessed the MCTS1-dependence of 17 genes underlying MSMD and other genes involved in IFN-γ immunity, including JAK2, by performing 5’UTR reporter assays in MCTS1^KO^ HeLa cells (Figure 4A). For many of these genes, we tested multiple alternative 5’UTRs. A significant decrease, of more than 65%, was observed only for the JAK2 reporter, which was rescued by the re-expression of WT MCTS1 but not the patients’ variants or the synthetic LOF allele A109D (Figure 4B). JAK2 reporter translation was also dependent on the presence of DENR (Figure S4A). We then investigated the molecular mechanism of the dependence of JAK2 translation on MCTS1. We identified three uORFs within the JAK2 5’UTR, two of which were ultra-short Start-Stop “stuORFs” (Figure 4C, Scheme of WT JAK2 5’UTR). Mutation of the uORFs within the JAK2 5’UTR revealed that the two stuORFs (uORFl and 2, but not uORF3) contributed to the MCTS1-dependence of this 5’UTR (Figure 4C). We also observed an accumulation of 40S ribosome footprints on both stuORFs in the endogenous JAK2 5’UTR (Figure 4D). We, thus, showed that the translation of JAK2, unlike that of 28 other genes related to IFN-γ immunity, was MCTS1-dependent. We then hypothesized that MCTS1 deficiency might impair JAK2 expression in MCTS1^KO^ cell lines and patients’ cells. CRISPR/Cas9-mediated knockout of MCTS1 in monocytic leukemia THP-1 cells caused a reproducible drop in endogenous JAK2 levels in five independent MCTS1^KO^ clones (Figure 5A, Antibody validation: Figure S4B). JAK2 production was also decreased, but not abolished, in P2’s SV40-fibroblasts, and this defect was rescued by transduction with WT MCTS1, but not with the patients’ variants or A109D (Figure 5B). Patient-derived T-saimiri virus-transformed T cells (HVS-T) and T-cell blasts had four-fold lower levels of JAK2 (Figure 5C–D). The re-expression of MCTS1 by stable transduction rescued JAK2 levels both in patients’ T-cell blasts (Figure S4C) and in MCTS1^KO^ THP-1 cells (Figure S4D). Thus, MCTS1 deficiency caused a three- to five-fold decrease in JAK2 protein levels in the three cell types tested (Figure 5E).

### Normal presence and frequency of leukocyte subsets in MCTS1 patients

JAK2 is one of four members of the Janus kinase family involved in JAK-STAT signaling in response to more than 50 cytokines^71^. No complete or partial genetic deficiency of human JAK2 has ever been reported and JAK2 knockout is embryonically lethal in mice^70^. Given the major hematological alterations observed in humans with JAK3^72^ deficiency (underlying SCID) and JAK2^KO^ mice, we hypothesized that impaired JAK2 expression in MCTS1-deficient patients would lead to abnormal myeloid or lymphoid development. However, MCTS1-deficient patients had normal complete blood count (CBC) profiles (Table S1). We also performed deep, mass cytometry-based immunophenotyping on four MCTS1-deficient patients (P2, P4, P5 and P7). We found that all the subsets detected (Figure S5A–D), including IFN-γ-producing lymphocyte subsets (Figure S5B) and IL-12- and IL-23-producing monocytes and dendritic cells (DCs) (Figure S5C), were present at counts and frequencies (data not shown) in the range of age-matched controls. This suggests that MSMD in MCTS1-deficient patients is not mediated by an absence or low frequency of any particular leukocyte cell subset, consistent with the clinical phenotype of isolated, as opposed to syndromic MSMD.

### *MCTS1 deficiency impairs IFN*-γ *production upon BCG infection* in vitro

As one of the four JAK kinases, JAK2 mediates responses to three cytokines related to IFN-γ-mediated immunity — IL-12, IL-23, and IFN-γ — through its association with the IL-12Rß2, IL-23R, and IFN-γR2 receptor chains and the mediation of signal transduction after cytokine binding^73–75^. The 20 known JAK2-dependent cytokines and the phenotypes of humans with genetic variants resulting in a deficiency of one of these cytokines or the corresponding receptor are listed in Table S6. We hypothesized that MCTS1 deficiency might impair IFN-γ circuit activation (i.e. the relevant leukocyte function) in response to mycobacterial infection by decreasing JAK2 levels. We tested this hypothesis by infecting fresh whole-blood cells from healthy travel controls and MCTS1-deficient patients with BCG and measuring IFN-γ production after 48 hours. Whole blood from patients P2 and P5 contained IFN-γ levels 13-fold lower than those of healthy travel controls (Figure S5F). IFN-γ production was only partially rescued by co-stimulating the patients’ cells with IL-12, suggesting that IL-12 is not the major contributor to this defect. The defect of IFN-γ production following stimulation with BCG alone was as profound as that in TYK2- and IL-12Rß1-deficient patients (Figure S5F). In summary, leukocytes from MCTS1-deficient patients have a severely impaired response to BCG infection *in vitro* in terms of IFN-γ production, despite their normal counts of cells from the lymphoid and myeloid subsets.

### Intact type I and type II interferon response pathways in MCTS1-deficient cells and blood from the patients

Based on the defective response of the patients’ blood cells to BCG and the role of JAK2 in the response to IFN-γ^70^, we hypothesized that the low JAK2 levels in the cells of MCTS1-deficient patients would impair their response to IFN-γ. However, we found that this was not the case. MCTS1^KO^ THP-1 cells responded normally to IFN-γ stimulation, as measured by the induction of pSTAT1 (Figure S6A) and *IRF1* mRNA (Figure S6B, C). MCTS1^KO^ THP-1 cells also displayed normal levels of STAT1 phosphorylation in response to IFN-α (Figure S6A). Transcriptome-wide pathway induction, detected by RNA-seq upon treatment with IFN-α and two doses of IFN-γ, was also intact in MCTS1^KO^ THP-1 cells (Figure S6D). The response to IFN-γ was normal in terms of STAT1 phosphorylation, as shown by the western blot of SV40-fibroblasts from P2 (Figure S6E) as was the response to IFN-α, as shown by the induction of *MX1* mRNA (Figure S6F). We then investigated the response to IFNs in primary cells from the patients. We studied the response to IFN-γ and IFN-α2 in monocyte-derived macrophages (MDMs), monocyte-derived dendritic cells (MDDCs), and monocyte-derived osteoclasts (MOCs) for P2. These cells responded normally to both IFNs, as shown by assessments of STAT1 phosphorylation (Figure S6G–I). Moreover, MDMs from P2 had normal transcriptional responses, as shown by RNA-seq (Figure S6J). We also stimulated fresh blood from patients P4, P5, and four healthy controls with IFN-γ and IFN-α, and used mass cytometry to assess the induction of STAT phosphorylation. The induction of STAT1, STAT3, and STAT5 phosphorylation was generally similar in patients and controls following treatment with IFN-α or IFN-γ, in all peripheral leukocyte subsets studied (Figure S6K). Two conclusions can be drawn from these findings. First, the low levels of JAK2 observed in MCTS1-deficient cells are not sufficiently low to impair responses to IFN-γ, either in MCTS1-deficient cell lines or in primary cells from patients. Second, even though *IFNAR1* is a reproducible MCTS1 target in both patient-derived SV40-fibroblasts and T-cell blasts (Figure 3F, G), the response to IFN-α is intact in MCTS1-deficient cell lines and primary cells from the patients. This finding is consistent with the absence of severe viral infections in the five MCTS1-deficient patients (Case Reports in Methods Section), despite exposure to common viruses, as attested by positive viral serology results for P2 and P5 (Figure S6L, M, Table S7). The patients’ cellular responses to IFN-γ and IFN-α were intact.

### Defective IL-23 and intact IL-12 response pathways in MCTS1-deficient cells

Given the intact cellular response to IFN-γ in the patients, we hypothesized that cellular responses to IL-12 and IL-23, which are also mediated by JAK2^73–75^, might be impaired in MCTS1-deficient patients. We used primary T-cell blasts from the patients to assess cellular responses to IL-12. These cells responded normally to IL-12 stimulation, as assessed by measuring the induction of pSTAT4 (Figure S7A). Patient-derived HSV-T cells also responded normally to IL-12 through the induction of *IFNG* mRNA (Figure S7B). Patient-derived or isogenic IL-23-sensitive cells are not available, because T-cell blasts and HSV-T cells do not display robustly detectable STAT3 phosphorylation upon IL-23 treatment in long-term culture (Figure S7C). We assessed the response to IL-23 and IL-12 in T-cell blasts by transcriptome profiling. T-cell blasts from nine healthy individuals, four MCTS1-deficient patients, two patients homozygous for the hypomorphic P1104A TYK2 allele that selectively impairs responses to IL-23^18^, and one patient each with autosomal recessive, complete TYK2, IL-12Rß1, and IRAK4 deficiencies were stimulated with IL-12, IL-23, IL-1ß, or IL-1ß plus IL-23 for 6 hours. We then performed RNA sequencing. We used IL-1ß as an IL-23-sensitizing agent, as previously described^35,42^. We found that MCTS1-deficient T-cell blasts responded normally to IL-12 and IL-1ß, but that their responses to IL-23 and to IL-23 plus IL-1ß were impaired (Figure 5G, Figure S7D). As MDMs apparently also respond weakly to IL-23 (Sun and Abraham, 2020) by inducing the expression of antimicrobial genes (e.g. p40phox and NOS2), we stimulated MDMs from three healthy controls and P2 with IL-23 for 6 hours. The response of MDMs from P2 to IL-23 was weak compared to healthy controls, as shown by pathway enrichment analysis on RNA-seq data, whereas these cells responded normally to IFNs (Figure S6J). Thus, the response to IL-12 was intact, whereas the response to IL-23 was impaired in the cells of MCTS1-deficient patients.

### Impaired JAK2 expression in MCTS1-deficient cells impairs cellular responses to IL-23

We then investigated whether the impaired response to IL-23 in MCTS1-deficient cells was due to low levels of JAK2 expression. We used a HEK-Blue IL-23 reporter cell line, in which the cells were stably transduced with IL-23R and a STAT3-dependent reporter gene. We produced polyclonal knockout lines for MCTS1 (MCTS1^KO^) or JAK2 (JAK2^KO^) by CRISPR/Cas9 gene editing (Figure S7E). The MCTS1^KO^ HEK cells had ~50% the normal level of JAK2 protein (Figure S7F), a deficit smaller than that observed in the patients’ cells (Figure 5E). As expected, JAK2^KO^ HEK-blue cells had a strongly impaired response to IL-23 (Figure S7G). We also found that MCTS1^KO^ cells had an impaired response to IL-23 at concentrations of both 1 ng/ml and 0.1 ng/ml (Figure S7G). We stably transduced these HEK-blue cells with a construct harboring an empty expression vector (EV) or a construct containing the MCTS1 coding sequence (M). MCTS1 overexpression rescued JAK2 expression in MCTS1^KO^ cells but not in JAK2^KO^ HEK-Blue cells (Figure 5G). Importantly, MCTS1 overexpression also rescued the impaired response to IL-23 of MCTS1^KO^ HEK-Blue cells, whereas it increased the response to IL-23 only slightly in the other three lines (Figure 5H). These findings confirm that the impaired response to IL-23 is due to the lack of MCTS1. Finally, we stably transduced these four HEK-Blue lines with an empty expression vector (EV) or a construct encoding the JAK2 ORF (J2) under the control of an endogenous promoter for the production of moderate amounts of JAK2. This partially rescued JAK2 levels in MCTS1^KO^ and JAK2^KO^ HEK-Blue cells (Figure 5I). Importantly, JAK2 overexpression rescued the response to IL-23 in MCTS1^KO^ and JAK2^KO^ HEK-Blue cells (Figure 5J). These findings demonstrate that a lack of MCTS1 impairs cellular responses to IL-23 through decreases in JAK2 expression.

### Particular dependence of the IL-23 response pathway on JAK2

We investigated the possibility of the IL-23 response pathway being particularly sensitive to decreases in JAK2 levels or activity. We transfected JAK2-deficient γ2A-fibrosarcoma cells expressing IL-23R and IL-12Rβ1 with various amounts of JAK2-encoding constructs and assessed their response to IL-23. In this system, the levels of IL-23-dependent STAT3 phosphorylation were dependent on JAK2 levels (Figure S7H). Thus, low levels of JAK2 protein can impair cellular responses to IL-23. We then investigated the dependence of several cytokine response pathways on JAK2. We expanded primary sorted NK cells by culture with feeder PBMCs and IL-2. These cells had a high purity (>95%, Figure S7I) and responded robustly to IL-23 plus IL-1β, or to IL-12, by secreting IFN-γ (Figure S7J). We also isolated primary monocytes, which responded to stimulation with IFN-α and/or IFN-γ by producing IP-10, IL-12p70 and/or TNF (Figure S7K–M). The response to IL-23 or IL-12 is mediated by JAK2 and TYK2, whereas the response to IFN-γ depends on JAK1 and JAK2 and the response to IFN-α depends on JAK1 and TYK2^77^. By contrast, the response to IL-1β is JAK2-independent, and even entirely independent of JAK proteins. We investigated the dependence of these cytokine response pathways on JAK2, using increasing concentrations of gandotinib, a JAK2-specific inhibitor (IC_50_ for JAK2=3nM, JAK1=19.8nM, TYK2=44nM, JAK3=48nM)^78^. We found that the response of NK cells to IL-23 + IL-1β was much more sensitive to gandotinib than the response to IL-12 (Figure 5K, Figure S7N). In monocytes, the responses to IFN-α and IFN-γ were as sensitive or less sensitive to gandotinib than the response to IL-12 (Figure 5K, Figure S7O,P,Q). Impaired or abolished cytokine responses can lead to higher cytokine concentrations in plasma, as observed in patients with IFN-γ or GM-CSF receptor deficiencies^79–81^. We hypothesized that impaired cytokine responses due to low JAK2 levels might increase cytokine concentrations in the plasma of MCTS1-deficient patients. We assessed the levels of 13 JAK2-dependent cytokines (Table S6) by bead-based ELISA. We found no detectable increase in plasma cytokine levels in the patients tested (Figure S7R). Finally, as another control, we tested the response to another JAK2-mediated cytokine, IL-27, in the patients’ T-cell blasts. We found a normal response to several concentrations of IL-27, in terms of STAT1 and STAT3 phosphorylation in T-cell blasts from patients, relative to healthy controls (Figure S7S,T). Thus, the biological response to IL-23 is tightly dependent on JAK2 levels, to a greater extent than responses to other JAK2-dependent cytokines, including IL-12, IL-27, and IFN-γ. These findings are consistent with the clinical phenotype of MCTS1-deficient patients being restricted to MSMD (Table S6).

### Defective IL-23 response pathway in the PBMCs of patients with MCTS1 deficiency

**Figure 6:**
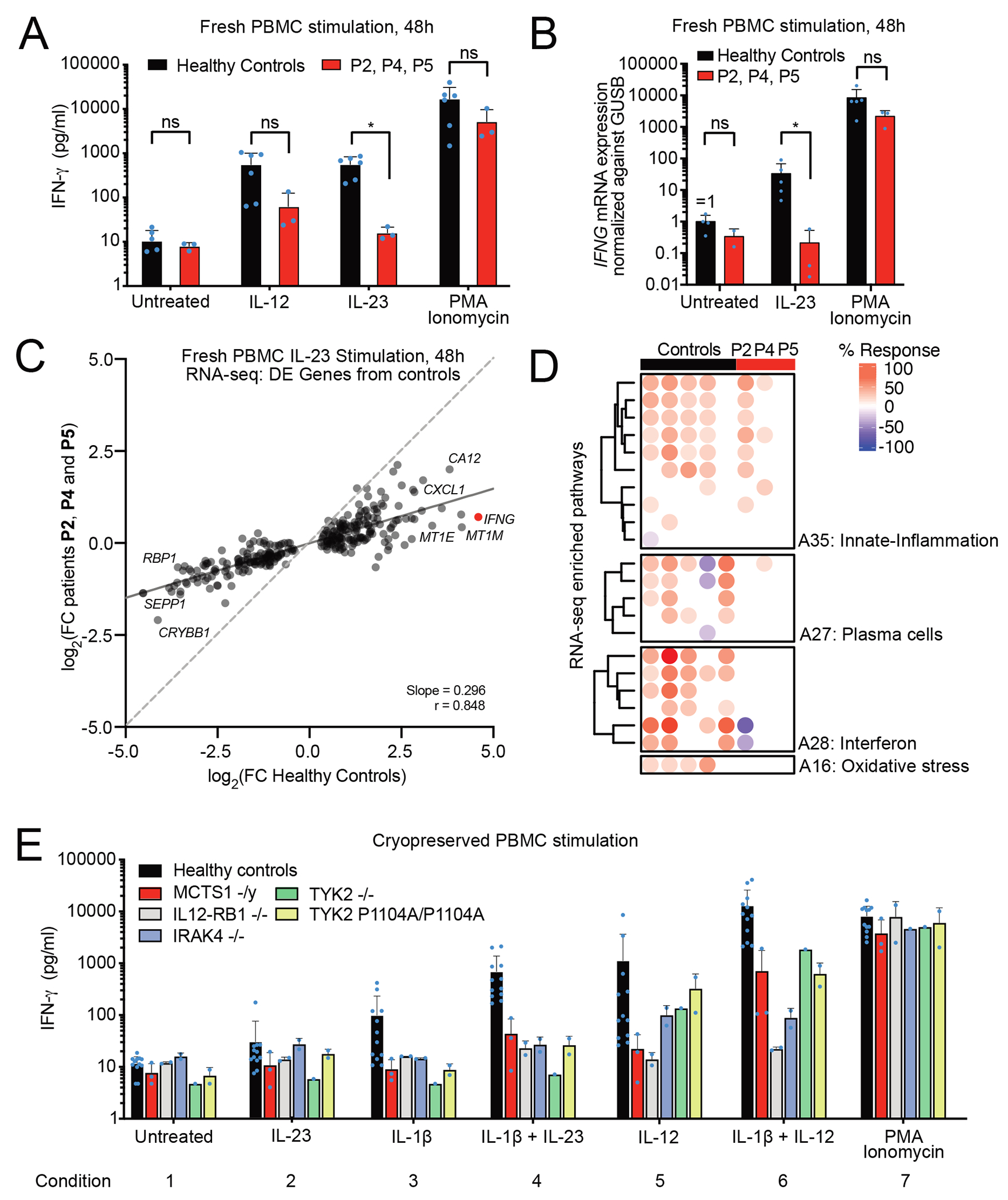
Defective IL-23-dependent IFN-γ induction in the patients’ primary immune cells **(A-B)** Fresh PBMCs were stimulated with 50 ng/ml IL-12 or 100 ng/ml IL-23 for 48 hours or with PMA plus ionomycin for 24 hours. IFN-γ levels in the supernatant were assessed by LEGENDplex multiplex ELISA **(A)** and *IFNG* mRNA induction was assessed by qPCR **(B)**. Statistical significance was assessed in unpaired Mann-Whitney *U* tests (*p<0.05). **(C)** RNA-seq analysis. Log_2_ fold-change in mRNA levels upon stimulation with IL-23 (100 ng/ml IL-23 for 48 hours) for transcripts differentially expressed in healthy controls, for the healthy controls and patients. **(D)** Heatmap showing the proportions of transcripts with changes in expression for indicated modules for the various individuals compared (controls versus P2, P4, P5) relative to baseline values in the absence of stimulation. Red: transcripts at higher levels than baseline, blue: decrease in transcript levels relative to baseline. **(E)** Cryopreserved PBMCs of the indicated genotypes were stimulated with 50 ng/ml IL-12, 100 ng/ml IL-23, 2.5 ng/ml IL-1β, or a combination of these cytokines for 48 hours, or with PMA + ionomycin for 24 hours. IFN-γ levels in the supernatant were assessed by LEGENDplex multiplex ELISA. Bars indicate the mean and SD.

Having shown that responses to IL-23 were impaired in the patients’ T-cell blasts, we investigated the responses of peripheral blood mononuclear cells (PBMCs) from the patients. We stimulated fresh PBMCs with IL-12, IL-23, or phorbol 12-myristate 13-acetate (PMA) plus ionomycin for 48 hours and measured IFN-γ levels in the medium. We found that levels of IFN-γ production by PBMCs from MCTS1-deficient patients were 10-fold lower after IL-12 treatment and 23-fold lower after IL-23 treatment than those of controls (Figure 6A), whereas IFN-γ production was normal after stimulation with PMA plus ionomycin. No *IFNG* mRNA induction upon IL-23 stimulation was observed in the PBMCs of MCTS1-deficient patients, whereas a 33-fold induction was observed in controls (Figure 6B), suggesting that this defect was not due to an impairment of *IFNG* mRNA translation. We also observed that the amounts of TNF produced in response to IL-23 stimulation were 23-fold lower in cells from patients than in those of controls (Figure S7U). For these samples, RNA sequencing revealed a significant induction of 291 genes upon stimulation with IL-23 in healthy controls, but no significant induction of any gene in MCTS-deficient patients (Table S8). An analysis of the correlation of the changes in expression of these 291 genes revealed a broadly impaired response to IL-23 in the PBMCs of MCTS1-deficient patients (Figure 6C). Modular repertoire analysis revealed that genes related to innate inflammation (inflammation, cytokines, monocytes, neutrophils), plasma cells, interferon and oxidative stress were poorly induced in the patients’ cells (Figure 6D), probably due to the combined low levels of stimulation of the IL-23 and IFN-γ response pathways, the latter being due to impaired IFN-γ production. Control cryopreserved PBMCs produced no detectable IFN-γ upon IL-23 stimulation (Figure 6E, condition 2) and required co-stimulation with IL-1ß to produce detectable amounts of IFN-γ upon IL-23 stimulation (Figure 6E, condition 4)^35^. Assessments of the response to IL-23 in the presence of IL-1ß also revealed a defect in cryopreserved PBMCs from MCTS1-deficient patients (Figure 6E, condition 3 versus condition 4). Cellular responses to IL-12 and IL-12 plus IL-1β were also partially impaired in the patients (Figure 6E, conditions 5 and 6). MCTS1-deficient PBMCs, thus, phenocopied PBMCs with *TYK2* mutations (*TYK2*^−/−^ and *TYK2* P1104A/P1104A) (Figure 6E). The patients’ PBMCs responded poorly to IL-23, with such stimulation failing, in particular, to induce IFN-γ.

### Impaired IFNG mRNA induction by IL-23 in Vδ2^+^ γδ T, MAIT and T_H_1* cells

**Figure 7:**
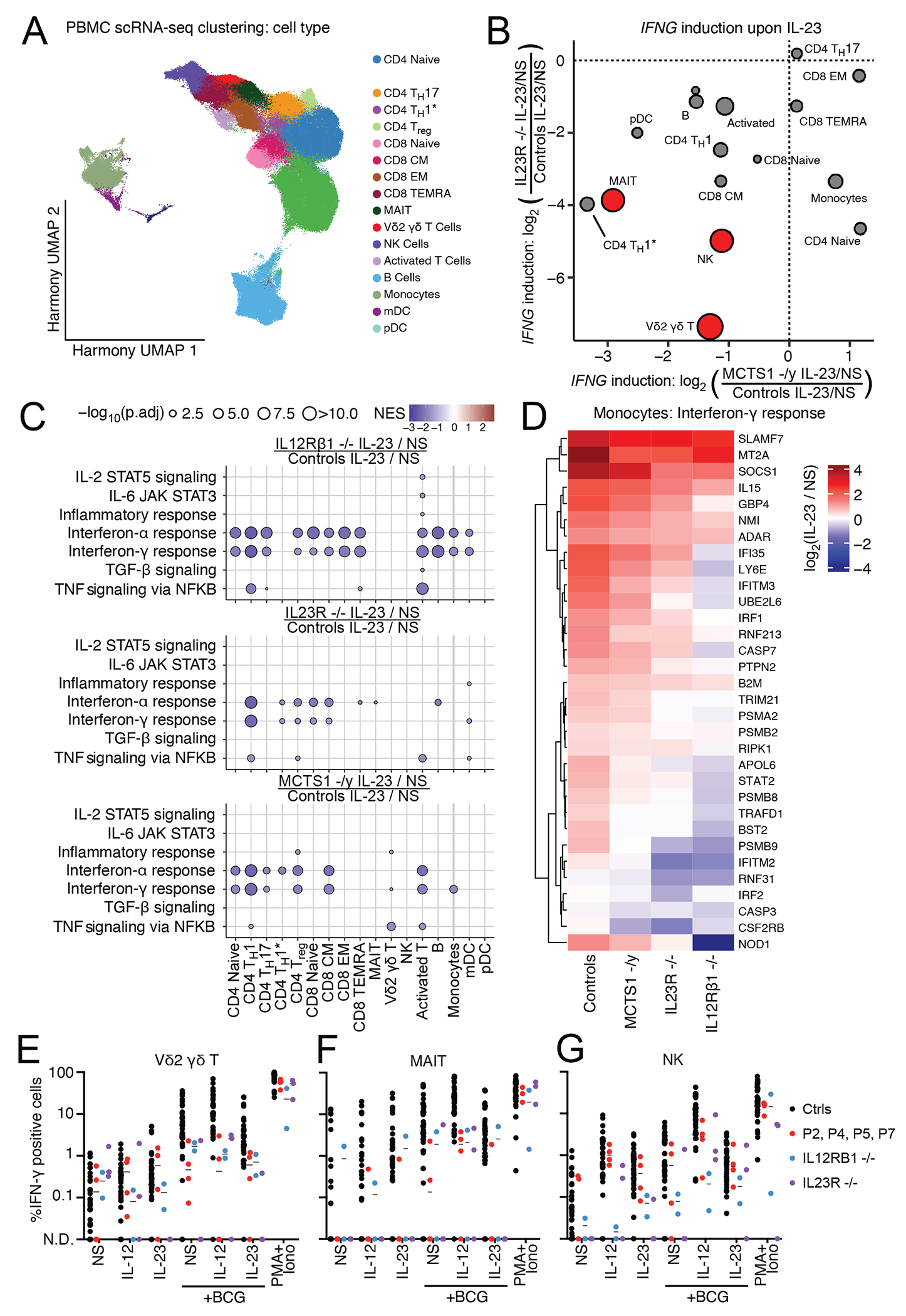
Patients’ Vδ2^+^ γδ T and MAIT cells have impaired *IFNG* induction upon IL-23 stimulation and BCG infection. **(A-D)** Single-cell RNA sequencing (scRNA-seq) analysis. PBMCs were either left non-stimulated or were stimulated with IL-23 for 6 hours, and single-cell capture was then performed. **(A)** Clustering analysis identifying 17 major leukocyte subsets. **(B)** The fold-change in *IFNG* mRNA levels following stimulation in MCTS1 patients relative to controls is shown on the *x*-axis. The *y*-axis shows the same parameter for IL-23R^−/−^ patients as a comparison. The size of the circles indicates the median change (IL-23 versus NS) in normalized *IFNG* mRNA levels in controls for the corresponding subset. **(C)** Pathway analysis of the transcriptional response to IL-23 in IL-12Rβ1^−/−^, IL-23R^−/−^ and MCTS1-deficient PBMCs relative to healthy control PBMCs, using the 50 Hallmark gene sets. Results are shown for selected immune-related gene sets. NES, normalized enrichment score. **(D)** Leading-edge genes for the Hallmark IFN-γ response gene set common to IL-12Rβ1-, IL-23R-and MCTS1-deficient patients relative to controls are shown. **(E-G)** Induction of IFN-γ in MCTS1-deficient Vδ2^+^ γδ T **(E),** MAIT **(F)** and NK **(G)** cells upon IL-23 stimulation and mycobacterial infection. Frequency of IFN-β-positive Vδ2^+^ γδ T cells following stimulation with cytokine, BCG or PMA plus ionomycin.

IFN-γ is mostly produced by T and NK lymphocytes^84^. Upon IL-23 stimulation, innate NK cells and innate-like adaptive Vδ2^+^ γδ T and MAIT cells are particularly potent producers of IFN-γ^30,42^. We hypothesized that some of these lymphocyte subsets might display impaired IFN-γ production in response to IL-23 stimulation in MCTS1-deficient patients. We tested this hypothesis, by stimulating cryopreserved PBMCs from seven healthy controls, four MCTS1-deficient patients, two IL-23R-deficient patients and one IL-12Rβ1-deficient patient with IL-23 for six hours and then performing single-cell RNA sequencing (scRNA-seq). This dataset incorporates several samples previously studied by Ogishi et al.^35^ and Philippot et al.^42^. After batch correction with Harmony and graph-based unsupervised clustering (Figure S8A–C), we were able to identify most PBMC leukocyte subsets (Figure 7A, Figure S8D–E). As previously reported, NK, Vδ2^+^ γδ T, and MAIT cells were the major subsets displaying *IFNG* induction upon IL-23 stimulation (Figure S8F). As reported for patients with IL-23R or IL-12ßR1 deficiency, MCTS1-deficient Vδ2^+^ γδ T and MAIT cells displayed impaired *IFNG* induction following IL-23 stimulation relative to healthy controls (Figure 7B, Figure S8G). The induction of *IFNG* expression was also impaired in purely adaptive Th1* cells, which normally produce less IFN-γ in response to IL-23 than innate-like T cells^42^. By contrast to IL-23R and IL-12Rβ1 deficiencies, the induction of *IFNG* by NK cells was moderately impaired in MCTS1-deficient patients (Figure 7B, Figure S8G). We also observed a broadly impaired transcriptional response to IL-23 stimulation in MCTS1-deficient PBMCs, resembling that seen in IL-12Rβ1- and IL-23R-deficient PBMCs (Figure 7C). This global transcriptional response probably represents a combination of the cellular responses to IL-23 and to the IFN-γ secreted in response to IL-23, as observed for example in monocytes following the stimulation of MCTS1-deficient PBMCs with IL-23 (Figure 7D). Overall, the response to IL-23 is impaired in the Vδ2^+^ γδ T, MAIT, and T_H_1* cells of MCTS1-deficient patients, leading to an impairment of *IFNG* mRNA induction by these cells in response to stimulation with IL-23.

### Impaired induction of IFN-γ secretion by IL-23 in Vδ2^+^ γδ T and MAIT cells

We then investigated the possibility that the impaired response to IL-23 in Vδ2^+^ γδ T and MAIT cells might impair the response of these cells to mycobacterial infection. We treated cryopreserved PBMCs with various stimuli, including the two cytokines (IL-12, IL-23), BCG, alone or with either or both cytokines, for 48 hours and assessed the amount of IFN-γ generated by these lymphocyte subsets by intracellular staining and flow cytometry. Consistent with our previous results, we found that IFN-γ production by Vδ2^+^ γδ T and MAIT cells upon stimulation with IL-23, BCG, or IL-23 plus BCG, was defective relative to that in healthy controls (Figure 7E–F). Interestingly, IFN-γ production was impaired only very mildly, if at all, in NK cells (Figure 7G). We investigated whether Vδ2^+^ γδ T and MAIT cells had a stronger defect of JAK2 expression than other T cells. We generated T-cell blasts from sorted CD4^+^, Vδ2^+^ γδ T, or MAIT cells (Figure S8H) and measured their JAK2 levels by western blotting. All T-cell blasts had low levels of JAK2 (Figure S8I), but the defect was not stronger in Vδ2^+^ γδ T-cell and MAIT blasts. Nevertheless, P2’s primary T-cell blasts displayed a stronger mean decrease in JAK2 expression than the primary myeloid cells of P2 (particularly MDMs and MOCs) (Figure S8J). This suggests that the specific impairment of the IL-23 response pathway and the partial impairment of the IL-12 response pathway in MCTS1-deficient patients could be explained in part by stronger JAK2 depletion in T lymphocytes than in myeloid cells. In summary, JAK2 expression and IL-23-dependent IFN-γ production are impaired in the Vδ2^+^ γδ T and MAIT cells of MCTS1-deficient patients.

## Discussion

We describe a surprising, new genetic etiology of MSMD: XR MCTS1 deficiency in six male individuals from five unrelated kindreds and four distant ancestries. Complete MCTS1 deficiency impairs ribosome recycling and translation reinitiation in the patients’ cells. JAK2 is one of about 150 human genes subject to MCTS1-mediated translation re-initiation. JAK2 protein levels in MCTS1-deficient myeloid and lymphoid cells are about three- to four-fold lower than those in control cells, with a greater decrease in levels observed in lymphoid than in myeloid cells. Responses to IFN-γ and IL-12, both of which normally require JAK2, were normal in these cells. By contrast, the response to IL-23 was impaired in the patients’ T-cell blasts and MCTS1 KO HEK Blue cells, which was rescued by exogenous expression of MCTS1 or JAK2. The patients MAIT and Vδ2^+^ γδ T-lymphocyte subsets produce only small amounts of IFN-γ in response to IL-23 and BCG. We show here that decreases in JAK2 levels or activity impair IL-23-dependent IFN-γ production by lymphocytes, providing a molecular and cellular basis for disease, and mechanistically and causally connecting the *MCTS1* genotype and the MSMD phenotype. Remarkably, an unbiased genome-wide forward genetics approach revealed an unexpected MSMD phenotype in patients with human MCTS1 deficiency.

Our finding is unexpected because MCTS1 is ubiquitously expressed and has a fundamental molecular function in the dissociation of deacylated tRNAs from post-termination 40S ribosomal complexes during ribosome recycling (on mORF stop codons) and re-initiation (on uORF stop codons). The translation re-initiation functions of DENR and MCTS1 are essentially identical in amplitude ^58,59,62^. However, DENR^KO^ is embryonically lethal in mice, whereas MCTS1^KO^ mice are viable ^60^. This is consistent with the more severe ribosome recycling defect of DENR^KO^ HeLa cells than of MCTS1^KO^ HeLa cells. Furthermore, we found no MSMD patient homozygous for rare candidate DENR variants. The apparent lack or extreme rarity of DENR-deficient patients suggests that this deficiency may be embryonically lethal in humans, as it is in mice. This contrasts with our findings that all five patients with MCTS1 deficiency have isolated MSMD and are otherwise healthy at ages of six months to 18 years. However, only one MCTS1-deficient patient has yet reached adulthood, and other clinical phenotypes may appear as the patients age.

We define the set of human MCTS1-dependent genes in cells from MCTS1-deficient patients. We identify JAK2 as a translational target of DENR-MCTS1 and probably the key contributor to MSMD. Decreases in the levels of other MCTS1-dependent proteins do not appear to have clinical consequences. Surprisingly, partial JAK2 deficiency impairs cellular responses to IL-23 and the subsequent induction of IFN-γ in lymphocytes, but not other cytokine response pathways involving JAK2. DENR^KO^ has been reported to impair JAK2 translation and subsequent responses to IFN-γ in mouse tumor cells^85^. Responses to IL-12 and IL-23 were not tested in this previous study. We found the same connection between MCTS1 and JAK2 in human cells, but the response to IFN-γ was intact. Low levels of JAK2 may have a much greater effect in IL-23- and IL-12-responsive cells than in IFN-γ-responsive cells. It is probably no coincidence that the four types of MSMD-causing *TYK2* variants, including the common P1104A variant, have an impact on the IL-23 response pathway, whereas only two of these types of variants also impair responses to IL-12^35^. This finding suggests that the IL-23R is more dependent on qualitative and quantitative variations of JAK2 or TYK2 than the IL-12R. Biochemical differences may be responsible for the weaker IL-23R signaling observed.

Our findings support our previous reports that human IL-23 is a key IFN-γ-inducing cytokine^18,30,42,73^. Despite having the p40 chain in common, IL-23 has historically been seen as an IL-17-inducing cytokine^73,88–91^, whereas IL-12 is seen as the key IFN-γ-inducing cytokine^73,91^. Recent discoveries have suggested that human IL-23 plays a key role in IFN-γ-mediated immunity to mycobacteria, and a more minor role in IL-17-mediated immunity to fungi. First, AR complete IL-23R deficiency underlies MSMD with almost complete clinical penetrance but only a mild form of CMC with incomplete penetrance^30,42^. Second, homozygosity for the common TYK2 P1104A allele selectively impairs responses to IL-23 and underlies MSMD with low penetrance and tuberculosis with high penetrance, but has no effect on susceptibility to CMC^18,54^. Third, impairment of the cellular response to IL-23 is the only mechanism underlying mycobacterial disease common to patients with the five forms of AR TYK2 deficiency^28,60^. Fourth, MCTS1-deficient patients have impaired cellular responses to IL-23 and isolated MSMD, with no detectable IL-17 deficiency or CMC. Collectively, these observations suggest that human IL-23 acts more as an IFNγ-inducing antimycobacterial cytokine than as an IL-17-inducing antifungal cytokine.

We found that IFN-γ induction in response to IL-23 stimulation was impaired in the Vδ2^+^ γδ T and MAIT cells of MCTS1-deficient patients. This finding is consistent with previous reports, as Vδ2^+^ γδ T and MAIT cells are among the subsets producing the largest amounts of IFN-γ in response to IL-23^30,42^. Furthermore, the IFN-γ production of these cells in response to IL-23 is impaired in IL-12Rβ1^−/−^, IL-23R^−/−^ and TYK2^−/−^ individuals^30,42,60^. NK cells are also potent inducers of IFN-γ upon stimulation with IL-23, but their transcriptional response to IL-23 is only moderately impaired in MCTS1-deficient patients. Interestingly, the induction of IFN-γ was also impaired in the patients’ Th1* cells. Th1* cells have a lower level of IFN-γ induction than MAIT, Vδ2^+^ γδ T and NK cells. Nonetheless, Th1* cells display more than an eight-fold induction of IFN-γ in response to IL-23 (Figure S8F), this induction being totally abolished in MCTS1-, IL-12Rβ1-, and IL-23R-deficient patients. Th1* cells are the major lymphocyte subset producing IFN-γ after exposure to BCG *in vitro*^92,93^. The counts of these cells in the blood increase in individuals with latent tuberculosis infection^94^. IL-23 may, thus, also contribute to Th1*-mediated immunity to mycobacteria.

Finally, we can speculate about the clinical phenotype of MCTS1-deficient patients in the absence of BCG vaccination. Surprisingly, none of the six MCTS1-deficient individuals had EM disease. This may be due to the protection afforded by BCG disease, as seen in IL-12Rß1-deficient patients^38,39^. It may also reflect an incomplete IFN-γ deficit, as only one of the seven reported IL-23R-deficient patients suffered from EM disease^41,42^ and cellular responses to IL-23 are impaired, but not abolished in MCTS1-deficient patients. In regions of endemic tuberculosis, in which BCG vaccination is not widely or efficiently performed, or if penetrance for BCG disease is incomplete, MCTS1 deficiency may be revealed by severe tuberculosis. Overall, this study reveals a surprising mechanistic connection between deficiency of a basic, ubiquitous, biochemical mechanism and a selective predisposition to mycobacterial disease.

### Limitations of the study

The five unrelated patients studied are all relatively young, between 1 and 18 years of age. We cannot therefore rule out the possibility of hitherto undetected clinical phenotypes other than MSMD arising as the patients age. We demonstrated that there is an impaired response to IL-23 in MCTS1^KO^ HEK-Blue cells and that this can be rescued by JAK2 overexpression. This suggests that the impaired IL-23 response in MCTS1 deficient patients is solely due to reduced levels of JAK2 expression. Yet, we have not, for technical limitations, carried out this rescue experiment in PBMCs of the patients and can therefore not completely rule out the possibility that other MCTS1 targets contribute to the phenotype *in vivo*.

## STAR METHODS

### RESOURCE AVAILABILITY

#### Lead contact

Further information and requests for resources and reagents should be directed to and will be fulfilled by the lead contact, Jonathan Bohlen (jonathan.bohlen@institutimagine.org).

#### Materials availability

All raw and processed data and biological materials, including immortalized cell lines from patients, are available upon request from the lead contact under a Material/Data Transfer Agreement with Inserm or the Rockefeller University.

#### Data and Code Availability

The RNA-seq, single-cell RNA-seq, and CITE-seq data have been deposited at SRA and are available at project ID PRJNA1004232. Custom software is available from GitHub: https://github.com/aurelioteleman/Teleman-Lab. The original western-blot images, flow cytometry data, mass spectrometry data, and microscopy data reported in this paper will be shared by the lead contact upon request.

Any additional information required to reanalyze the data reported in this paper can be obtained from the lead contact upon request.

This paper does not report original code.

### EXPERIMENTAL MODEL AND STUDY PARTICIPANT DETAILS

The age, sex, ancestry and race of the studied patients is reported in the main text of the paper and in the method details below. Information on gender and socioeconomic status of the patients was not collected. All patients are male because we study an X-linked recessive genetic disease. Informed consent was obtained in the countries of residence of the patients, in accordance with local regulations (kindred A in China, kindred B in Finland, kindred C in Saudi Arabia and kindreds D and E in Iran) and with institutional review board (IRB) approval. Experiments were conducted in France, Germany, Qatar, and the United States of America, in accordance with local regulations and with the approval of the IRB of the Rockefeller University (protocol no. JCA-0699) and INSERM (protocol no. C10-07 and C10-16) and by the Institutional Research Ethics Boards of Sidra Medicine and Qatar Biobank, for the United States of America, France and Qatar, respectively. Healthy controls were recruited in Finland, France, Iran, Saudi Arabia, and the United States of America.

### METHODS DETAILS

#### Human patients

Informed consent was obtained in the countries of residence of the patients, in accordance with local regulations (kindred A in China, kindred B in Finland, kindred C in Saudi Arabia and kindreds D and E in Iran) and with institutional review board (IRB) approval. A detailed questionnaire was completed by the physicians caring for the patients, including demographic data, clinical features, and biological and microbiological results, and the data were sent to J.Bo., Q.Zha. and J.Bu. A detailed clinical case report is provided below.

#### Case reports

##### Kindred A, P1.

P1 was the third child of nonconsanguineous parents of Chinese origin, with two healthy older sisters (Table S1, Figure 1A). He was born at full term in 2017, with no perinatal complications. He received the BCG vaccine (Danish strain) on the day after delivery. He was well until seven months of age, when an enlarged left axillary lymph node that was erythematous and tender was detected. Surgical incision and drainage were performed, but the local lesions relapsed. The lymph node under the mandibular region was also found to be enlarged. At the age of 14 months, P1 had recurrent fever associated with hepatosplenomegaly, chest wall abscess and osteomyelitis with multiple bone lesions. A computerized tomography (CT) scan showed an enlarged lymph node under the left armpit, with necrosis, multiple sublobe atelectasis in the left lung, multiple abscesses in the spleen, lesions in the ribs on both sides of the body, multiple vertebrae, and the right ilium. Hepatosplenomegaly was confirmed by CT-scan. Acid-fast bacilli (AFB) were detected in the abscess and disseminated mycobacterial infection (BCG-osis) was diagnosed. The whole metagenome of the abscess was shotgun-sequenced, and *Mycobacterium bovis* BCG was detected. Antimycobacterial treatment was implemented, based on isoniazid (INH), rifampin (RIF), ethambutol (EMB), amikacin, and linezolid; subcutaneous treatment with recombinant IFN-γ was also initiated. However, the patient’s medical condition deteriorated, and the patient died in 2019.

##### Kindred B, P2.

P2 is a boy originating from and living in Finland (Table S1, Figure 1A). He was born in 2003 and received the BCG vaccine (Danish strain) at birth. Three months later, he developed BCG-itis consisting of an enlarged lymph node in the left groin. On ultrasound examination, intra-abdominal lymph nodes were detected, with para-iliac and para-aortic extension. One lymph node was excised and cultured, yielding *M. bovis* BCG. P2 progressively developed a high fever, tiredness and poor appetite, and a diagnosis of disseminated BCG-osis was established. Treatment with INH, RIF, EMB, ciprofloxacin, amikacin and IFN-γ was initiated and the patient recovered well. Treatment with four antimycobacterial drugs (excluding amikacin) and IFN-γ was continued for one year, and drug treatment was then gradually stopped. When P2 was two years and nine months old and on RIF/INH, the symptoms recurred, a severe relapse was diagnosed and treatment with five drugs plus IFN-γ was reinstated. At the age of five years, P2 was well, with no symptoms, a normal chest X ray and abdominal ultrasound results, and was treated with RIF/INH/IFN-γ twice weekly. At his most recent consultations (in 2019 and 2021), he was well, without treatment. No other severe infections have occurred. P2 has a healthy sister who is growing normally. P2 received all other routinely indicated vaccines without complication. At five years of age, he was assessed by a neurologist for learning difficulties, but no neurological issues were identified and magnetic resonance imaging (MRI) was not indicated. At the age of seven years, a CT scan of the head, thorax and upper abdomen showed no abnormalities. Abdominal ultrasound was performed yearly until the age of nine years, and all the organs detected were of normal size and development. At the age of 16 years, the patient consulted an endocrinologist as he was slightly overweight. Thyrotropin-releasing hormone (TRH) stimulation test results and the levels of sex hormones, T3 and TSH were all normal. T4V levels were slightly below the lower limit of the normal range. The patient was therefore considered healthy. He swims, skis, and rides horses and motorbikes.

##### Kindred C, P3.

P3 is a boy originating from and living in Saudi Arabia (Table S1, Figure 1A). He was born in 2010 and received the BCG vaccine (Danish strain) at birth. At the age of one year, he underwent excision of the left axillary lymph node due to BCG-itis. At the age of three years, he was referred to the hospital for painful swelling of the distal third of the left leg of six months’ duration. An X ray of the left leg showed an intramedullary lytic lesion with surrounding cortical thickening at the diaphysis of the distal tibia, and magnetic resonance imaging (MRI) results were suggestive of chronic osteomyelitis, with a lesion in the distal tibia diaphysis measuring 5.2 cm x 1.1 cm associated with an extensive periosteal reaction. A biopsy was performed on the left tibia; histological examination revealed inflammatory granulation, and *M. bovis* BCG susceptible to INH, RIF, and EMB and resistant to pyrazinamide (PZA) grew in culture. P3 was treated with INH, RIF, and clarithromycin for 12 months, with an excellent clinical response resulting in total resolution. Immunophenotyping results for T, B and NK cells, immunoglobulin levels (IgG, IgM and IgA) and oxidative burst assay (in neutrophils) results were normal. The patient is currently 12 years old, alive and well, and performing well at school. He presented no unusual infections during follow-up and was fully immunized according to the usual schedule, with no adverse effects. P3 has four sisters and one brother, who is 18 years old and healthy, and, like the sisters, presented no complications after BCG vaccination or severe/atypical infections.

##### Kindred D, P4.

P4 is a boy, born in 2016 to nonconsanguineous parents from Iran (Table S1, Figure 1A). He received the BCG vaccine (Pasteur 1173p2 strain) at birth. He was hospitalized at the age of five months for right axillary lymphadenopathy. He developed an abscess with pus discharge in this area. An ultrasound scan of the axillary area revealed an internal echogenic mass (4.2 x 3.4 x 2.7 cm) and another hypoechoic node (3.7 x 3.3 x 1.5 cm). PCR results were positive for *M. tuberculosis* complex and culture confirmed *M. bovis* BCG infection. P4 received INH, EMB, RIF and levofloxacin supplemented with folic acid and vitamins, but the parents stopped the treatment after four months. Nine months later, P4 suffered another right axillary lymphadenopathy with pus discharge and fever. PCR was positive for *M. tuberculosis* complex. The patient received treatment with INH, EMB and clarithromycin for nine months. Abdominal ultrasound results and a bone scan were normal. P4 presented no infection with any other organism and was in good health, except for his low Ig levels. He is currently in good health, on monthly IVIG treatment for his low Ig levels. He has no siblings, and there is no history of IEI in his relatives and cousins. His mother had a history of abortion due to the absence of a fetal heartbeat and a neural tube defect at 16 weeks of pregnancy. At the age of five months, P4 underwent abdominal ultrasound and abdominopelvic CT scans. All the organs detected were of normal size and development. P4 is now seven years old. He is physically active, plays football and socializes with his peers. His growth is normal, and abdominal and pelvic ultrasound scans show the detected organs (including the liver, spleen, bile ducts, gallbladder, pancreas, kidneys and urinary bladder) to be normal in size and development.

##### Kindred E, P5, P6 and P7.

P5 was born to consanguineous Iranian parents in 2019 (Table S1, Figure 1A). He was vaccinated with BCG (Pasteur 1173P2 strain) at birth. At the age of three months, he presented a left axillary draining adenopathy, which was resected surgically at seven months. Histological analysis of the lymph node showed an epithelioid granuloma containing AFB. The presence of BCG was documented by PCR. P5 was treated with RIF and INH for six months. At 13 months of age, he developed ascites, multiple abdominal adenopathies, and hepatic calcified nodules. Ascitic fluid analysis revealed a lymphocytic exudate. Liver biopsy showed cirrhosis, but no mycobacterial elements were documented. Bone marrow biopsy results were normal. P5 received antimycobacterial treatment with RIF, INH, EMB, ofloxacin and clarithromycin for nine months. He is currently well without treatment, with mild hepatosplenomegaly but no other symptoms. P5 has a sister who is well and has never been hospitalized, and a brother (**P7**) who was identified as a mutation carrier a few days after birth and was therefore not vaccinated with BCG; P7 has no history of mycobacterial infection. The height and weight of P5 and P7 have been routinely monitored and no abnormalities have been found. P5 and P7 also had an uncle (**P6**), the brother of their mother, who died at the age of two years from fever and hepatosplenomegaly. P6 was vaccinated with BCG (Pasteur 1173P2 strain) at birth.

#### WES and Sanger sequencing

For P1, genomic DNA (gDNA) was extracted with the Qiagen blood mini kit, and DNA fragments were generated and enriched for whole-exome sequencing with the Agilent SureSelect XT Human All Exon V5 kit. Sequencing was performed on an Illumina HiSeq X10 apparatus (Illumina). Reads were mapped on the human reference genome ^95^ with Burrows-Wheeler aligner-0.7.12 ^96^. Variant calling was then performed on the aligned reads, with GATK-3.4-46 best practices ^97^. For annotation of the variants, we used SnpEff-4.1a ^98^ tools. For P2, P3 and P4, gDNA was isolated from whole blood with the iPrep PureLink gDNA Blood Kit and iPrep Instruments (Life Technologies, Thermo Fisher Scientific). Exome capture was performed with the SureSelect Human All Exon 50 Mb kit (Agilent Technologies), with 3 μg gDNA. Single-end sequencing was performed on an Illumina Genome Analyzer IIx (Illumina). For P5, gDNA was isolated from whole blood. WES was performed at Macrogen Korea (Macrogen, Inc., Seoul, South Korea). For WES, exon enrichment was performed with the SureSelect XT Library Prep Kit (Agilent Technologies, CA, USA). Sequencing was performed on a NovaSeq 6000 (Illumina, CA, USA) sequencer generating 150 bp reads, with 100× coverage. The NGS platform generally covered >95% of the targeted regions with up to 99% sensitivity.

All variants from the patients and their familial segregation (when appropriate materials were available for analysis) were confirmed by amplifying the flanking regions of the variants obtained with specific primers (Figure S1A–E). PCR products were analyzed by electrophoresis in 1% agarose gels, sequenced with the Big Dye Terminator v3.1 cycle sequencing kit (Applied Biosystems), and analyzed on an ABI Prism 3700 sequencer (Applied Biosystems).

#### Enrichment analysis

We performed an enrichment analysis for pLOF variants of the *MCTS1* gene on our cohort of 422 male patients with MSMD of unidentified genetic etiology. As controls, we used 1,932 male individuals of diverse ethnic origins from our in-house database. These controls were patients with various genetically unexplained non-mycobacterial infections other than chronic mucocutaneous candidiasis (CMC). We used the Firth penalized logistic regression method ^99^, as implemented in the R (v4.1) ^100^ package logistf, to compare the proportion of individuals with pLOF variants between the MSMD and control groups. The analysis was adjusted for the first five principal components (PCs) to account for the ethnic heterogeneity of the samples. Principal component analysis (PCA) was performed with Plink v1.9 software on WES data, with the 1000 Genomes (1kG) Project phase 3 public database as a reference, for 2,942 exonic variants with minor allele frequency (MAF) > 1% and a call rate > 99%.

#### Exon trapping assay

A DNA segment encompassing the *MCTS1* exon 2 and 3 region (ChrX: 120604950 to ChrX: 120606870 region, GRCh38 reference, release 13) was amplified from genomic DNA extracted from whole-blood samples from a healthy control. It was inserted between the *Xho*I and *Bam*HI sites of the pSPL3 vector. The sequence was then mutated to encode the variant of P3. Plasmids containing wild-type (WT) and mutant *MCTS1* exon 2-3 regions were then used to transfect COS-7 cells. After 24 hours, total RNA was extracted and reverse-transcribed. The cDNA products were amplified with primers binding to the flanking HIV-TAT sequences of the pSPL3 vector and ligated into the pCR^™^4-TOPO^®^ vector (Invitrogen). Stellar^™^ cells (Takara) were transformed with the resulting plasmids. Colony PCR and sequencing with primers binding to the flanking HIV-TAT sequences of pSPL3 were performed to assess the splicing products transcribed from the WT and mutant alleles.

#### Patient-derived cell lines

*Herpesvirus saimiri*-transformed T (HVS-T) cells were generated with the TERT transformation system. HVS-T cells were cultured in 40% RPMI, 40% Panserin 401, 20% fetal-calf serum (FCS) supplemented with 10 ng/ml IL-2, 50 μg/ml gentamycin (Gibco, 15750060) and GlutaMAX^™^ (Gibco, 35050061). T-cell blasts (T-blasts) were induced from 2 million PBMCs (fresh or cryopreserved) in 2 ml ImmunoCult^™^-XF T Cell Expansion Medium (Stem Cell Technologies, 10981) supplemented with 10 ng/ml IL-2 (Gibco, PHC0023) and 1:40 diluted ImmunoCult^™^ Human CD3/CD28/CD2 T Cell Activator. T-blasts were then cultured in ImmunoCult medium with IL-2 for 2-3 weeks, before restimulation in the same manner. Dermal primary fibroblasts were obtained from skin biopsy specimens, transformed with SV40 antigen and cultured in DMEM (Gibco) supplemented with 10% FCS. All cell lines used tested negative for mycoplasma.

#### Cell-line culture

We cultured HeLa cells, HEK293T cells, γ2A- fibrosarcoma cells and HT1080 cells in DMEM +10% FCS +100 U/ml penicillin/streptomycin (Gibco 15140122). We subcultured the cells with trypsin-EDTA. HeLa cells tested negative for mycoplasma and were authenticated by SNP typing. THP-1 cells were cultured in RPMI supplemented with 10% FCS.

#### Translation reporter assays

HeLa cells were used to seed 96-well plates at a density of 8,000 cells per well. Cells were transfected for 16-20 hours in the presence of Lipofectamine 2000, with 100 ng *Renilla* luciferase plasmid and 100 ng firefly luciferase plasmid per well. For translation reporter assays including MCTS1 reconstitution conditions, we used 60 ng of EV, *MCTS1* WT or variant expression plasmid, 70 ng of *Renilla* luciferase reporter plasmid and 70 ng of firefly reporter plasmid per well. Four hours after transfection, the medium was replaced. All the *Renilla* luciferase plasmids contained the 5’UTR of interest, whereas all the firefly luciferase plasmids contained the MCTS1-independent *Lamin B1* 5’UTR and were used as a transfection normalization control. We added 0.4 μl Lipofectamine reagent to each well. Cell transfection was always performed with technical triplicates. Luciferase activity was assessed 16-20 hours after transfection, with the Promega Dual-Luciferase^®^ Reporter Assay System, in accordance with the manufacturer’s instructions.

#### 40S and 80S ribosome footprinting

We performed 40S and 80S ribosome footprinting, as previously described ^65,101^. Briefly, HeLa cells and SV40-fibroblasts were grown to ~80% confluence in multiple 15 cm dishes (2-3 dishes per treatment). The cells were briefly washed with 1x PBS, 10 mM MgCl_2_, and 400 μM cycloheximide, and freshly prepared crosslinking solution (1x PBS, 10 mM MgCl_2_, 400 μM cycloheximide, 0.025% PFA, 0.5 mM DSP) was added. Cells were incubated with crosslinking solution for 15 minutes at room temperature with slow rocking. The crosslinking solution was then poured off and the remaining crosslinker was inactivated by incubation for 5 minutes with ice-cold quenching solution (1x PBS, 10 mM MgCl_2_, 200 μM cycloheximide, 300 mM glycine). The quenching solution was poured off and 150 μl lysis buffer (0.25 M HEPES pH 7.5, 50 mM MgCl_2_, 1 M KCl, 5% NP40, 1000 μM cycloheximide) was added to each 15 cm dish. The cells were incubated at 4°C for two minutes for lysis. The cell debris was scraped off the dish and the lysate was collected. The lysates were briefly vortexed and clarified by centrifugation at 20,000 x *g* for 10 minutes at 4°C. The supernatant was collected and its RNA concentration was determined with a Nanodrop spectrophotometer. We then added 100 U RNAse 1 (Ambion, AM2294) per 120 μg of RNA. Lysates were incubated for 5 minutes at 4°C and then loaded onto 17.5%-50% sucrose gradients and centrifuged for 5 hours at 35,000 rpm in a Beckman Ultracentrifuge equipped with an SW40 rotor. Gradients were fractionated with a Biocomp Gradient Profiler system. We collected the 40S and 80S fractions for immunoprecipitation and footprint isolation. The 40S and 80S fractions corresponding to one or two 15 cm dishes were used for the direct extraction of RNA for total footprint samples. The crosslinker was then removed and RNA was extracted from the footprint fractions: 55 μl (1/9 volume) of crosslink-removal solution (10% SDS, 100 mM EDTA, 50 mM DTT) and 600 μl acid-phenol chloroform (Ambion) were added and the mixture was incubated at 65°C, with shaking at 1,300 rpm for 45 minutes. The tubes were then placed on ice for 5 minutes and centrifuged for 5 minutes at 20,000 x *g*. The supernatant was washed once with acid-phenol chloroform and twice with chloroform, and the RNA was then precipitated with isopropanol and used for library preparation (see below). The precipitated or total proteins were isolated from the organic phase. We added 300 μl ethanol and 1.5 ml isopropanol and the solutions were incubated at −20°C for 1 hour. Proteins were sedimented by centrifugation at 20,000 x *g* for 20 minutes, washed twice with 95% ethanol and 0.3 M guanidine-HCl, dried and resuspended in 1x Laemmli buffer.

#### Deep-sequencing library preparation

The quality and integrity of the 40S and 80S RNA extracted were determined on an Agilent Bioanalyzer with the total RNA Nano 6000 Chip. For size selection, RNA was subjected to electrophoresis in 15% urea-polyacrylamide gels (Invitrogen) and fragments of 20-80 nt (40S) or 25-35 nt (80S) in size were excised, with the Agilent small RNA ladder used as a reference. RNA was extracted from the gel pieces by smashing the gels into small pieces with gel smasher tubes and extracting the RNA in 0.5 ml 10 mM Tris pH 7 at 70°C for 10 minutes. The gel pieces were removed and RNA was precipitated in isopropanol. The RNA was then dephosphorylated by incubation with T4 PNK (NEB, M0201S) for 2 hours at 37°C in PNK buffer without ATP. It was precipitated again and purified in isopropanol. Footprints were then analyzed with an Agilent Bioanalyzer small-RNA chip and the Qubit smRNA kit. We used up to 25 ng of footprint RNA as input for library preparation with the SMARTer smRNA-SeqKit for Illumina from Takara/Clontech Laboratories, according to the manufacturer’s instructions. For RNA-seq libraries, total cell RNA was extracted with TRIzol and libraries were prepared with the Illumina TruSeq stranded library preparation kit. Deep-sequencing libraries were sequenced on the Illumina Next-Seq 550 system.

#### Data analysis and statistics

Analysis of ribosome footprinting NGS data: Adapter poly-A sequence nucleotides were trimmed from raw reads with cutadapt (https://doi.org/10.14806/ej.17.1.200). Ribosomal RNA and tRNA reads were removed by bowtie2 alignment with human tRNA and rRNA sequences ^102^. The remaining reads were then separately aligned with the human transcriptome (Ensembl transcript assembly 94) and the human genome (hg38) with BBmap (sourceforge.net/projects/bbmap/). Counted reads were generally normalized by sequencing depth (number of alignments per library). Reads were counted, and metagene plots and single-transcript traces were obtained with custom software written in C and available from GitHub (https://github.com/aurelioteleman/Teleman-Lab). Translation efficiency (TE) was calculated from the number of 80S ribosome footprints in a coding sequence divided by the number of RNA sequencing reads on the coding sequence. uORFs were annotated on the basis of the presence of an ATG and an inframe stop codon within the 5’UTR of any mRNA. Only uORFs beginning with an ATG were considered.

#### Stable lentiviral transduction

Lentiviral particles were produced in HEK293T cells by transfection with pPAX2, pHBX2, pVSV-G and pTRIP EV or plasmids containing the *MCTS1* variants, with X-tremeGENE 9 (Roche, 6365779001). The medium was replaced after five hours. The supernatant was harvested 20 hours post-transfection, filtered, supplemented with 8 μg/ml protamine sulfate and added, in a 1:1 volumetric ratio, to 150,000 cells in 100 μl medium. Cells were spinoculated for 2 hours at 1200 x *g*. The cells were cultured for three to four days, assayed for transgene expression by FACS staining for ΔNGFR, and subjected to enrichment for MACS sorting.

#### Generation of MCTS1 and JAK2 knockouts in THP-1 cells and HEK-Blue IL-23 cells

THP-1 cells or HEK-Blue IL-23 cells were transduced with pLENTI-V2 constructs encoding Cas9 and an sgRNA (GCCTGGGAAGGATACAACAA) targeting the *MCTS1* gene and an sgRNA targeting *JAK2* (GGTAAGAATGTCTTGTAGCT). Cells were then selected by culture with 5 μg/ml (THP-1) or 1 μg/ml (HEK-Blue) puromycin for two weeks. The KO pool was then tested for efficient knockout by western blotting, and seeding was performed by limiting dilution for clonal expansion. Clones were screened by western blotting and *MCTS1* knockout was confirmed by gDNA sequencing.

#### Immunoblotting

Cells were lysed in standard RIPA lysis buffer containing protease inhibitors (Roche mini EDTA-free, 1 tablet in 10 ml) and phosphatase inhibitors (2 mM sodium Ortho-vanadate, Roche Phosstop 1 tablet in 10 ml, 0.1 M sodium fluoride, 0.1 M beta-glycerophosphate) and Benzonase (50 U/ml), after brief washing in FCS-free DMEM or PBS. Lysates were clarified and protein concentration was determined in a BCA or Bradford assay. Equal amounts of protein were subjected to SDS-PAGE and the resulting bands were transferred to a nitrocellulose membrane with 0.2 μm pores. Membranes were subjected to Ponceau staining, incubated in 5% skim milk in PBST for 1 hour, briefly rinsed with PBST and then incubated in primary antibody solution (5% BSA in PBST or 5% skim milk in PBST) overnight at 4°C. Membranes were then washed three times, for 15 minutes each, in PBST, incubated in secondary antibody solution (1:5,000 in 5% skim milk in PBST) for 1 hour at room temperature, then washed again three times, for 15 minutes each, in PBST. Finally, chemiluminescence was detected with ECL reagents and a Biorad Chemidoc. The membranes were not stripped. The antibodies used in this study are listed in the key resources table.

#### RT-qPCR

Total RNA was isolated from cells with the Quick-RNA MicroPrep spin column kit (Zymogen, R1050), according to the manufacturer’s protocol. For cDNA synthesis, we used 10 ng of total RNA for random oligomer-primed reverse transcription with Superscript II Reverse Transcriptase (Invitrogen, 18064022). Quantitative RT-PCR was carried out with the *Taq*Man Universal Master Mix (Applied Biosystems, 4305719) and *Taq*man probes for *GUSB* (4326320E), *IRF1* (Hs00971965_m1), *IFNG* (Hs00989291_m1) and *MX1* (Hs00895608_m1; Thermo Fisher Scientific).

#### Deep immunophenotyping of primary leukocytes by mass cytometry (CyTOF)

CyTOF was performed on whole blood treated with heparin (1.0 × 10^6^ cells per panel), with the Maxpar Direct Immune Profiling Assay (Fluidigm), according to the manufacturer’s instructions. Cells were frozen at −80°C after overnight dead-cell staining. Acquisition was performed on a Helios machine (Fluidigm). All the samples were processed within 24 hours of sampling. Data were analyzed with OMIQ software.

#### Monocyte isolation and differentiation into MDMs, MDDCs and osteoclasts

CD14^+^ monocytes were isolated from fresh PBMCs with CD14 MicroBeads (Miltenyi Biotec, 130-050-201) according to the manufacturer’s instructions. For the induction of monocyte-derived macrophages (MDMs), CD14^+^ monocytes were cultured in RPMI 1640 supplemented with 10% FCS, 1% penicillin/streptomycin and 50 ng/ml recombinant human (rh)M-CSF (R&D Systems, 216-MC) for 7 days, and were then allowed to differentiate for seven days in the presence of 50 ng/ml rhM-CSF and 50 ng/ml rhIL-4 (R&D Systems, 204-IL). Monocyte-derived dendritic cells (MDDCs) were induced by culturing CD14^+^ monocytes for 7 days in RPMI 1640 supplemented with 10% FCS, 1% penicillin/streptomycin, 50 ng/ml rhGM-CSF (R&D Systems, 215-GM) and 20 ng/ml rhIL-13 (R&D Systems, 213-ILB). For osteoclast differentiation, CD14^+^ cells were cultured in *α*-MEM (Thermo Fisher Scientific, 12571063) containing 10% FCS, 1% penicillin/streptomycin, 30 ng/ml rhM-CSF and 10 ng/ml rhRANKL (Peprotech, 310-01) for 7 days. The medium was replaced every three days.

#### Cell-line cytokine stimulation and flow cytometry

Cell lines were cultured as described above. For stimulation, cells were starved of IL-2 and 2% FCS overnight, as appropriate. They were then stimulated with the indicated cytokine concentrations for the indicated times at 37°C. Subsequently, for qPCR, total RNA was extracted and subjected to reverse transcription, as described above. Alternatively, dead cells were removed by Live/Dead Aqua (Thermo Fisher Scientific, L34966) staining for 5 minutes at 4°C, and the cells were then fixed and permeabilized for FACS analysis with BD Fix Buffer I and Perm Buffer III. The cells were then stained with antibodies against pSTAT1 (BD, 612564), pSTAT4 (BD, 562073) or pSTAT3 (BD, 612569) and fluorescence was assessed on a Beckman Gallios Cytometer.

#### T-cell blast cytokine stimulation and RNA sequencing

T-cell blasts were starved of IL-2 overnight. They were then stimulated with 50 ng/ml IL-12 (R&D Systems, CAT: 219-IL-005) or 100 ng/ml IL-23 (R&D Systems, Cat: 1290-IL) for 6 hours or with 2.5 ng/ml IL-1ß (R&D Systems, CAT: 201-LB-005) for 24 hours. Double-stimulated cells were stimulated with IL-1ß for 18 hours and then with IL-23 and IL-1ß for 6 hours. Total RNA was isolated from cells with the Quick-RNA MicroPrep spin-column kit (Zymogen, R1050), according to the manufacturer’s protocol. RNA was then subjected to RNA-seq library preparation and deep-sequencing at the Rockefeller University Genomics Core Facility.

#### The HEK-Blue IL-23 sensitive cytokine stimulation and colorimetric assay

HEK-Blue assays were performed in accordance with the manufacturer’s instructions. Briefly, HEK-Blue cells were maintained under selection with the supplier’s selection compound mixture at a dilution of 1:250. For cytokine stimulations, cells were used to seed 96-well plates at a density of 25,200 cells per well, in 200 μl stimulation medium containing either without cytokines or containing 5 ng/ml IL-12, 1 ng/ml IL-23, 0.1 ng/ml IL-23 or 10.000 IU/ml IFN-α2a. After incubation for 20 hours, 20 μl culture supernatant was mixed with 180 μl QUANTI-Blue solution, incubated for 30 minutes at 37°C and then its optical density at 620-655 nm was then measured on a photometric plate reader.

#### Monocyte and NK cell sorting, culture and stimulation

NK cells were sorted from fresh PBMCs with a BD Aria II, SORP cytometer and CD56^+^ CD3^−^ negative live cells were selected. This cell population was then expanded by culture in the presence of a 10-fold excess of PBMCs from the same donor irradiated with 35 grays in ImmunoCult^™^-XF T Cell Expansion Medium (Stem Cell Technologies, 10981) supplemented with 10 ng/ml IL-2 (Gibco, PHC0023) and 8% FCS. Before stimulation, the cells were starved of FCS and IL-2 for 20 hours. They were incubated with the indicated concentration of gandotinib for 1 hour and then treated with 100 ng/ml IL-23, 2.5 ng/ml IL-1β and/or 50 ng/ml IL-12. The purity of the NK cell preparation was assessed by flow cytometry, as illustrated in Figure S7F. Monocytes were sorted from fresh PBMCs with Miltenyi Biotec anti-CD14 magnetic microbeads in accordance with the manufacturer’s instructions. They were transferred to RPMI supplemented with 10% FCS, and 1% penicillin/streptomycin and stimulated, on the same day, with gandotinib and cytokines, as described above for NK cells. IFN-α2a and IFN-γ were both used at a concentration of 10,000 IU/ml. After 24 hours, supernatants were collected and assayed by LEGENDplex bead-based ELISA.

#### PBMC stimulation

PBMCs were isolated with Lymphoprep (StemCell, 07801), from blood drawn no more than 48 hours previously into lithium heparin tubes, in accordance with the manufacturers’ instructions. PBMCs were then either cryopreserved in 90% FCS, 10% DMSO or used fresh. For PBMC stimulation (fresh or cryopreserved), 100,000 PBMCs were used to seed each well of a round-bottomed 96-well plate, in RPMI supplemented with 10% human serum. They were stimulated for 48 hours with 2.5 ng/ml IL-1ß, 50 ng/ml IL-12, 100 ng/ml IL-23, 0.1 v/v% BCG or a combination of these stimuli. We added PMA (Sigma CAT: P1585-1MG, 8 ng/mL) + ionomycin (Sigma CAT: 56092-81-0, 10^−5^ M) for the last 24 hours only. The supernatants and cell pellets were collected. Supernatants were subjected to LEGENDplex multiplex ELISA with Human Inflammation Panel 1 (BioLegend, 740809), according to the manufacturer’s instructions. Total RNA was extracted from the cell pellets for qPCR and RNA-seq analysis, as described below.

#### RNA sequencing analysis

Total RNA sequencing was performed with an Illumina NovaSeq S2 flowcell (read length 100 bp) with a read depth of 30 M. All FASTQ sequences passed quality control tests and were aligned with the GRCh38 reference genome with STAR (2.6.1d). BAM files were converted to a raw count expression matrix with featurecount. Raw count data were normalized with DEseq2. The ensemble ID targeting multiple genes was collapsed (average) and a final gene data matrix was used for a modular repertoire analysis as previously described ^82,83^ or for gene set enrichment analysis (GSEA: fgsea) with hallmark gene sets (http://www.gsea-msigdb.org/).

#### Protein isolation from human polymorphonuclear neutrophils

Polymorphonuclear neutrophils were isolated by density gradient centrifugation (Lymphoprep, Stem Cell Technologies) and red blood cells were lysed by incubation in red cell lysis buffer (8.02 g NH_4_Cl, 0.84 g NaHCO_3_, 0.37 g EDTA in 1 L H_2_O, pH 7.4) at 37°C for 15 min. Proteins were isolated with modified RIPA buffer (25 mM Tris–HCl pH 7.4, 150 mM NaCl, 1% NP-40 and 1 mM EDTA) supplemented with DTT (0.1 mM, Thermo Fisher Scientific), protease inhibitor cocktail (Merck), phosphatase inhibitor cocktail (Merck), PMSF (1 mM, Merck) and DFP (0.1 mM, Merck).

#### Whole-blood activation and ELISA for cytokines

Venous blood samples from healthy (local and travel) controls and patients were collected into heparin-containing collection tubes. These samples were diluted 1:2 in RPMI 1640 (GibcoBRL) supplemented with 100 IU/ml penicillin and 100 μg/ml streptomycin (GibcoBRL). We then dispensed 1 ml of each diluted blood sample into each of five wells (1 ml/well) of a 48-well plate (Nunc), which was incubated for 48 hours at 37°C, under an atmosphere containing 5% CO_2_/ 95% air. The activation conditions were: medium alone, live BCG (*M. bovis*-BCG, Pasteur substrain) at a MOI of 20 BCG cells/leukocyte, BCG plus recombinant (rh) IL-12 (20 ng/ml; R&D Systems), or BCG plus IFN-γ (Imukin). The supernatants were collected after 48 hours and subjected to ELISA with the human IFN-γ, IL-12p40 (R&D) and IL-12p70 (R&D) ELISA kits, according to the manufacturer’s instructions.

#### VirScan - phage immunoprecipitation sequencing (PhIP-Seq)

Antibody profiling by phage immunoprecipitation sequencing (PhIP-Seq) was performed on plasma samples from patients and controls with an expanded version of the original VirScan library, and the data were analyzed as previously described ^26,103–105^, but with the following modifications. We calculated species-specific significance cutoff values to estimate the minimum number of enriched, non-homologous peptides required to consider a sample seropositive, as previously described with an in-house dataset and a generalized linear model ^106,107^. For each sample, we calculated virus-specific scores, by dividing the counts of enriched, non-homologous peptides by the estimated cutoff score. These adjusted virus scores are depicted on heatmap plots. We randomly selected an age-matched subset of 19 individuals from a larger cohort of 800 individuals of Arab ancestry (representing the general adult population) from our in-house database, for comparison with the patients and initial controls. Pooled human plasma used for IVIg (Privigen^®^ CSL Behring AG), and human IgG-depleted serum (Molecular Innovations, Inc.) were used as additional controls.

#### Single-cell RNA sequencing

For scRNASeq analysis following stimulation, we analyzed cryopreserved PBMCs from six healthy controls, two MCTS1-deficient patients, two IL-23R-deficient patients and one IL-12Rβ1-deficient patient, as previously described ^35,42^. Briefly, we filtered cells through a MACS SmartStrainer with 70 μm pores (Miltenyi Biotec, Cat: 130-098-462) to remove large debris, washed them three times with PBS plus 0.5% FCS, and finally filtered them through a Falcon Cell Strainer with 40 μm pores (Corning, Cat: 352340), before subjecting them to single-cell capture with the 10X Genomics Chromium chip. We prepared libraries with the Chromium Single Cell 3’ Reagent Kit (v3 Chemistry) and sequenced them with an Illumina NovaSeq 6000 sequencer. We preprocessed sequences with CellRanger. We sequenced about 10,000 cells per sample. We filtered data manually on the basis of common quality-control metrics. For cell-type identification, after integration with Harmony ^108^ to eliminate batch-, genotype-, and stimulation-dependent variation, we performed two sequential graph-based clustering analyses. The first round of clustering identified general leukocyte subsets, whereas the second round identified memory and effector T-lymphocyte subsets and NK lymphocytes at sufficiently high resolution. We identified clusters with the SingleR pipeline ^109^ guided by the reference RNA-Seq dataset generated by Monaco et al. ^110^, and cell type-specific marker gene expression was then assessed manually. We visualized clusters by uniform manifold approximation and projection (UMAP). We quantified gene expression at the single-cell level with Seurat ^111^. Pseudobulk analysis was performed by aggregating all reads from cells assigned to a given cluster, as previously described ^112^. We performed differential expression analysis with DESeq2 ^113^. We conducted geneset enrichment analysis (GSEA) with the fgsea package, by projecting the fold-change ranking onto various MSigDB genesets (http://www.gsea-msigdb.org/gsea/msigdb/genesets.jsp). All analyses were performed in R v.4 (http://www.R-project.org/). Raw data will be made available from SRA BioProject upon publication of the manuscript.

#### Stimulation of PBMCs with IL-12, IL-23, BCG and PMA/ionomycin for intracellular staining and flow cytometry analysis

This experiment was performed as previously described ^34^. Healthy adult donors were recruited at the Rockefeller University (New York, USA) and Necker Hospital (Paris, France). We plated 300,000 cells per well in 96-well U-bottomed plates, at a density of 1.5 to 3 x 10^6^ cells/ml. Cells were stimulated with IL-12 (50 ng/ml), IL-23 (100 ng/ml) and/or BCG (MOI of 1) for 41 hours. For PMA plus ionomycin treatment, cells were cultured for 40 h in RPMI. They were then stimulated with 50 ng/mL PMA and 1 mM ionomycin. After 1 hour, Golgiplug was added to all cultures, in accordance with the manufacturer’s protocol. Seven hours later, the cells were harvested for staining and flow cytometry. Cells were first stained with the Zombie NIR Viability kit (BioLegend) for 15 minutes. They were then stained with FcBlock (Miltenyi Biotec), anti-gdTCR-Alexa 647 (BioLegend), anti-CD3-V450 (BD Biosciences), anti-CD56-BV605 (BioLegend), anti-CD4-BUV563 (BD Biosciences), anti-Vd1TCR-FITC (Miltenyi Biotec), anti-CD8-BUV737 (BD Biosciences), anti-Vd2TCR-APC/Fire750 (BioLegend), anti-CD20-BV785 (BioLegend), anti-Va7.2-Alexa 700 (BioLegend), MR1-5-OP-RU-tetramer (NIH tetramer core facility), anti-Vb11-APC (Miltenyi Biotec), and anti-iNKT-BV480 (BD Biosciences) antibodies for 30 minutes. The stained cells were fixed with the FOXP3/Transcription Factor kit (Thermo Fisher Scientific) and subjected to intracellular staining with anti-T-bet-PE/Cy7 (BioLegend), anti-IFN-g-BV711 (BioLegend), anti-TNF-a-BV510 (BioLegend), anti-IL-17A-PERCP/Cy5.5, anti-RORgT-PE (BD Biosciences), anti-CD3-V450 (BD Biosciences), anti-CD4-BUV563 (BD Biosciences) and anti-CD8-BUV737 (BD Biosciences) antibodies in Perm/Wash buffer. Cells were then analyzed in a CyTek Aurora spectral flow cytometer.

### QUANTIFICATION AND STATISTICAL ANALYSIS

Appropriate statistical analyses were chosen depending on the experimental setting, number of replicates and type of data. The type of statistical test is always indicated in the figure legends of figure panels where statistical tests were performed. Generally, R suite was used to calculate statistical significance. All error bards in this paper display standard deviations (SD). No standard error of mean (SEM) is shown. Typically p < 0.05 was used as a threshold to determine statistical significance, this is always indicated in the figure legends.

## Supplementary Material

1 **Figure S1. Confirmation of patient variants and ethnicity, related to** Figure 1. **(A-E)** Sanger sequencing traces for probands (P1-P5) and their relatives. **(F)** PCA plot for patients P1-P5 and control data from the 1000 Genomes project generated from the WES data for the patients.

2 **Figure S2. Exon trapping for the P3 variant demonstrating aberrant splicing and an IGV snapshot of P4 and P5 WES data, related to** Figure 2. **(A)** Exon-trapping results for *MCTS1* exons 2 and 3, for the wild-type and P3 sequences. The region from the *MCTS1* gene is indicated by a blue line, whereas the region from the plasmid backbone is shown in black. **(B-C)** Aberrant *MCTS1* splicing in whole blood from patient 3. **(B)** Counts of reads mapping to junctions between exons 1-4 and intron 2 of the *MCTS1* mRNA in patient 3 and his parents. **(C)** Sashimi plot illustrating the splicing events observed in patient 3 and his parents. **(D)** Large deletion affecting exons 5 and 6 of *MCTS1* in P4 and P5. IGV snapshot of WES reads aligned with the *MCTS1* gene for data from P4, P5 and a control. **(E)** Amino-acid sequence alignment for MCTS2P and MCTS1. **(F-H)** Counts of RNA-seq reads aligned with *MCTS1* and *MCTS2P* for **(F)** whole-blood samples from P3 and his family, **(G)** MCTS1^KO^ and WT THP-1 cells, and **(H)** T-cell blasts of the indicated genotypes. **(I)** MCTS2P is hypomorphic for MCTS1-dependent reinitiation. Translation reporter assay on HeLa WT and MCTS1 KO cells. Cells were transfected with the indicated construct and reporter activity was measured. Columns and error bars indicate the mean and SD.

3 **Figure S3. Defective ribosome recycling and translation reinitiation in the patients’ cells, related to** Figure 3. **(A)** Summary of the experimental setup for 40S and 80S ribosome footprinting in SV40-fibroblasts. SV40-fibroblasts from P2, stably transfected with empty vector (EV) or wild-type (WT) MCTS1 were subjected to standard RNA-seq. In parallel, the cells were crosslinked and lysed. Lysates were treated with RNase 1 to obtain ribosome-protected mRNA fragments (footprints). The ribosomes were then separated on a sucrose gradient by density centrifugation, RNA was extracted from ribosome-containing fractions and deep-sequencing libraries of the ribosome footprints were prepared. **(B)** Western blot of the MCTS1 and tubulin proteins in the samples used for ribosome footprinting on WT and MCTS1^KO^ HeLa cells. Data from two independent experiments are shown. **(C-D)** Metagene profile of 40S **(C)** and 80S **(D)** ribosome footprints from control and MCTS1-knockout HeLa cells showing the position of the 5’ end of the ribosome footprints relative to the start codons. Read counts were normalized by sequencing depth. “Smooth” indicates that the curve was smoothed with a 3 nt sliding window. **(E)** Western blot of the MCTS1, DENR and tubulin proteins in the samples used for ribosome footprinting of P2’s SV40 fibroblasts +/− MCTS1 transduction. **(F-G)** Metagene profile of 40S **(F)** and 80S **(G)** ribosome footprints from SV40-fibroblasts from P2 transduced with empty vector (EV) or *MCTS1*, showing the positions of the 5’ ends of the ribosome footprints relative to the start codons. Read counts were normalized against sequencing depth. “Smooth” indicates the curve was smoothed with a 3 nt sliding window. **(H)** Metagene profiles for 40S ribosome footprints showing the positions of the 5’ ends of the ribosome footprints relative to uORF stop codons in SV40-fibroblasts. Read counts were normalized against sequencing depth. **(I)** The ribosome recycling defect is dependent on the penultimate codon of the uORFs. Penultimate codon enrichments for uORFs in the top quartile for 40S accumulation (*n* = 1048) relative to all detected uORF stop codons (*n* = 4230). Blue indicates significant enrichment, whereas red indicates significant depletion in the top quartile. Significance was assessed in binomial tests adjusted for multiple testing. **(J-K)** Penultimate stop codon enrichments for mORF **(J)** and uORF **(K)** stop codons in P2’s SV40-fibroblasts versus DENR^KO^ HeLa cells. **(L)** Correlation of differences in translation efficiency between MCTS1-deficient T-cell blasts and DENR^KO^ HeLa cells. Genes that are significantly (*p*<0.1) down-regulated in both datasets are shown in red. **(M-N)** Differences in translational efficiency **(M)** and RNA levels **(N)** of genes related to the IFN-γ circuit between T-cell blasts from patients (in red) and controls (in black). Data are normalized against healthy controls for each gene.

4 **Figure S4. Antibody-based confirmation of the presence of JAK2 protein and JAK2 expression in patient-derived cells upon rescue transduction, related to** Figures 4 and 5. **(A)** JAK2 translation is dependent on DENR. Efficiency of JAK2 reporter translation in HeLa cells transfected with siRNA targeting GFP or DENR, as assessed in dual-luciferase assays. Results are shown for three technical replicates representative of three biological replicates. **(B)** Western blot on total protein extracts from control (WT) and JAK^−/−^ HT1080 fibrosarcoma cells for the JAK2, MCTS1 and GAPDH proteins. **(C)** Western blot of T-cell blasts stably transduced with empty vector (EV) or *MCTS1*. Whole-cell lysates of T-cell blast lines from 1-2 healthy controls (HC), P2, P5, an IRAK4-deficient and an IL-12Rβ1-deficient patient were probed for JAK2, MCTS1, DENR and GAPDH. **(D)** Western blot of WT and MCTS1^KO^ THP-1 cells stably transduced with empty vector (EV), *MCTS1* or *JAK2*. Western blots were performed for JAK2, MCTS1 and DENR and the membrane was stained with Ponceau solution for the detection of all proteins present.

5 **Figure S5. Immunophenotyping of MCTS1-deficient patients, related to** Figure 5. **(A)** Deep immunophenotyping by mass cytometry of NK-cell subsets in healthy adults, healthy children and patients (P2, P4, P5, P7). **(B)** Deep immunophenotyping by mass cytometry of T-cell subsets. **(C)** Deep immunophenotyping by mass cytometry of dendritic-cell and monocyte subsets. **(D)** Deep immunophenotyping by mass cytometry of B-cell subsets. **(A-D)** No significant differences could be found between controls and the MCTS1-deficient patients in multiple *t*-tests incorporating correction for multiple tests. **(E)** Normal frequency of MAIT cells in MCTS1-deficient patients. The frequency of CD161^+^ TCRVα7.2^+^ MAIT cells as a percentage of CD3^+^ T cells was determined by flow cytometry in healthy controls, P2, P4 and P5 and two IL23R-deficient patients. **(F)** The patients’ leukocytes fail to produce sufficient IFN-γ upon infection with mycobacteria. ELISA analysis of IFN-γ levels in whole blood after stimulation with BCG, or BCG plus IL-12, in travel controls (historical and daily shown separately), MCTS1-deficient (P2 and P5), TYK2-deficient and IL-12Rβ1-deficient patients. UT, unstimulated. Statistical significance was assessed in Mann-Whitney tests, *p<0.05.

6 **Figure S6. Normal responses to IFN-γ and IFN-α in MCTS1-deficient cells and blood from the patients, related to** Figure 5. **(A)** Response of WT and MCTS1^KO^ THP-1 cells to IFN-γ. Cells were stimulated with IFN-γ or IFN-α2b at the indicated dose for 15 minutes and then fixed, stained for pSTAT1 and analyzed by flow cytometry. **(B)** Response of WT and MCTS1^KO^ THP-1 cells to IFN-γ. Cells were stimulated with IFN-γ or IFN-α at the indicated dose for 16 hours, and total RNA was then extracted and gene expression was assessed by RT-qPCR. **(C)** Western blot of the THP-1 MCTS1 polyclonal KO pool. Western blots were performed for MCTS1 and vinculin. **(D-E)** Intact IFN-γ and IFN-α2b responses in MCTS1KO THP-1 cells. Analysis of differentially expressed genes (DEGs) in THP-1 cells treated with IFN-α or IFNγ for 6 hours. The IFN-α2b and IFN-γ treatment conditions were compared to non-stimulated conditions. Gene set enrichment analysis (GSEA) was based on the fold-change in ranking against the Hallmark (HM) gene sets (http://www.gsea-msigdb.org/gsea/msigdb/genesets.jsp?collection=H). **(D)** The top 10 HM pathways are shown on a dot heatmap. Red colors indicate that the normalized enrichment score (NES) of transcripts for a given gene set mostly increased, whereas blue colors indicate a predominant decrease in enrichment scores relative to non-stimulation conditions. Dot size indicates −log_10_FDR (false discovery rate). **(E)** Response of the SV40 fibroblasts of patient P2 and a control (HC) to IFN-γ. Cells were left unstimulated (US) or were stimulated with IFN-γ at the indicated dose for the indicated time. Whole-cell lysate was then obtained and subjected to western blotting. **(F)** Response of SV40-fibroblasts from P2 and a control, and of fibroblasts from IFNAR1-, IFNAR2-, STAT2- and IRF9-deficient patients to IFN-α. Cells were stimulated with IFN-α at the indicated dose or were left unstimulated (US) for eight hours, and levels of mRNA for *MX1* relative to *GUSB* were then determined by RT-qPCR. The data shown were obtained in four independent experiments. **(G-I)** Intact response to interferons in the patients’ primary myeloid cells. MDMs (G), MDDCs (H) and osteoclasts (I) from three healthy controls and P2 were derived from primary monocytes by differentiation protocols outlined in the methods section. These cells were stimulated with the indicated concentrations of IFN for 30 minutes and were then lysed and analyzed by western blotting. **(J)** MDMs were stimulated with the indicated cytokines for 6 hours and total RNA was analyzed by RNA-sequencing. Analysis of differentially expressed genes (DEGs) in the indicated conditions relative to non-stimulated cells. Gene set enrichment analysis (GSEA) was based on the fold-change in ranking against the Hallmark (HM) gene sets. **(K)** The patients’ primary leukocytes respond normally to IFN-α2b and IFN-γ. Fresh blood from P4 and P5 and three healthy controls was treated with IFN-α2b or IFN-γ for 20 minutes and then subjected to mass cytometry as described in the methods section. STAT phosphorylation was assessed by determining the log_2_ fold-change in mean fluorescence intensity (MFI) between non-stimulated and stimulated conditions for each individual. Mean values are shown on a color-coded scale. **(K)** Viral serological results for P2, P4 (under IgG IV), P5 and P7. **(L)** The pathogen-specific antibodies in P4 and his mother are similar to those found in healthy children. Normalized detection of pathogen-specific antibodies by VirScan. P4 was under IgG IV at the time of this sampling.

7 **Figure S7. Responses to IL-12 and IL-23 in MCTS1-deficient cells and PBMCs, related to** Figures 5 and 6. **(A)** Responses of T-cell blasts from patients (P2 and P5) and healthy controls to IL-12. Cells were stimulated with IL-12 or IFN-α2b at the indicated dose for 15 minutes and were then fixed, stained for pSTAT4 and analyzed by flow cytometry. **(B)** Response of patient (P2) and control HSV-T cells (healthy controls and IL-12Rβ1^−/−^) to IL-12. Cells were stimulated with IL-12 or IFN-α at the indicated dose for 4 hours; total RNA was then extracted and gene expression was assessed by RT-qPCR. **(C)** Response of healthy control T-cell blasts to IL-23. Cells were stimulated with IL-23 or IFN-α at the indicated dose for 15 minutes and were then fixed, stained for pSTAT3 and analyzed by flow cytometry. **(D)** Impaired response to IL-23 in MCTS1-deficient T-cell blasts. Heatmap representation of the log_2_ fold-change (stimulation vs. non-stimulation) in expression for the MYC Target V2 Hallmark gene sets. Rows represent individual genes and columns represent samples grouped by stimulus, with the expression profiles of individual subjects shown for each set of conditions. **(E-F)** Western blot **(E)** of MCTS1KO and JAK2KO HEK-Blue Il-23 sensitive cells and quantification **(F)** of JAK2 levels from three biological replicates of the western blot. **(G)** Cytokine stimulation of HEK-Blue Il-23 sensitive cells. Cells were stimulated for 20 hours with the indicated cytokines and enzymatic reporter activity was assessed according to the manufacturer’s instructions. The plot shows four biological replicates with statistical significance assessed in paired, two-tailed *t*-tests, *p<0.05. **(H)** JAK2 is rate-limiting for the IL-23 response. JAK2-deficient γ2A^−^ fibrosarcoma cells stably expressing IL-23R and IL-12Rβ1 were transiently transfected with various amounts of plasmid encoding JAK2, diluted against a plasmid encoding GFP. The cells were then stimulated with 10 ng/ml IL-23 for 30 minutes and harvested for western blotting. HT1080 fibrosarcoma cells were used to determine endogenous JAK2 protein levels. **(I)** The expanded NK cell population is pure. Cultured NK cells were analyzed by flow cytometry after two weeks of culture. **(J-M)** Responses of primary NK cells and monocytes to cytokine stimulation as determined by assessing cytokine secretion. **(J)** Cultured NK cells were starved of IL-2 for 24 hours and were then stimulated for 24 hours with the indicate cytokines. **(K,L,M)** Monocytes were isolated from fresh PBMCs by magnetic sorting for CD14^+^ and were directly stimulated with the indicated cytokines for 24 hours. Culture supernatants were analyzed by bead-based ELISA. **(N)** Cultured NK cells were stimulated with the indicated concentration of gandotinib, with the addition of IL-12 or IL-1β + IL-23 after 1 hour and further incubation for 24 hours. Supernatants were analyzed by bead-based ELISA. (**O,P,Q**) Sorted monocytes were stimulated with the indicated concentration of gandotinib, with the addition of IFN-α or IFN-γ after one hour and further incubation for 24 hours. Supernatants were analyzed by bead-based ELISA. **(R)** Cytokine levels in the plasma of MCTS1-deficient patients P2, P4 and P5 and healthy, ethnically matched controls. Cytokine levels were assessed by LEGENDplex bead-based ELISA. **(S, T)** Quantification of the response to IFN-α and IL-27 in T-cell blasts from controls and patients by staining for **(S)** pSTATl and **(T)** pSTAT3 and flow cytometry. Statistical significance was assessed in unpaired, two-tailed non-parametric *t*-tests; no significant differences were observed, ns = not significant. **(U)** The patients’ PBMCs do not secrete TNF in response to IL-23 stimulation. Fresh PBMCs from patients (in red) and heathy controls (WT/WT, in black) were stimulated with 100 ng/ml IL-23 for 48 hours or with PMA plus ionomycin for 24 hours. TNF production was assessed by subjecting the supernatant to LEGENDplex multiplex ELISA. Statistical significance was assessed in unpaired, two-tailed non-parametric *t*-tests, *p<0.05, ns = not significant.

8 **Figure S8. Single-cell transcriptomic analysis of MCTS1-deficient leukocytes, related to** Figure 7. **(A-C)** Harmonized UMAPs for scRNA-seq data color-coded for batch **(A)**, genotype **(B)** and stimulation **(C)**, showing appropriate integration with respect to the corresponding variables. **(D)** Expression levels of characteristic marker genes in the indicated leukocyte subsets, confirming appropriate subset attribution. **(E)** Leukocyte subset frequency in cryopreserved PBMCs from healthy controls (ctrls), IL-23R-, IL-12Rβ1- and MCTS-deficient patients. **(F)** Normalized IFN-γ expression in healthy control leukocyte subsets in non-stimulated and IL-23-stimulated conditions. **(G)** *IFNG* expression is poorly induced by IL-23 stimulation in MCTS1-deficient patients’ Vδ2^+^ γδ T, MAIT and Th1* cells. The fold-change in *IFNG* mRNA levels following stimulation in MCTS1 patients relative to that in controls is shown on the *x*-axis. The *y*-axis shows the same parameter for IL-12Rβ1-deficient patients for comparison. The size of the circles indicates the median change (IL-23 versus NS) in normalized *IFNG* mRNA levels in controls for the corresponding subset. **(H)** Flow-cytometry analysis of cell type-specific T-cell blasts from healthy controls, P2 and an IL-12Rß1-deficient patient. **(I)** JAK2 levels in patients’ innate-like T-cell blasts. T-cell blasts induced from the sorted T-cell populations indicated, analyzed by western blotting. **(J)** Summary of JAK2 levels in patients’ lymphoid and myeloid cells. JAK2 levels were determined by western blotting and normalized against loading controls. Columns and error bars represent the mean and SD, respectively.

9 **Table S3, related to** figure 3**:** Genes differentially expressed in P2 SV40-fibroblasts upon MCTS1 transduction

10 **Table S5, related to** figure 3**:** Genes differentially expressed in the T-cell blasts of patients (*n* = 4) relative to controls (*n* = 4)

11 **Table S8, related to** figure 6**:** RNA-seq of PBMC stimulated with IL-23.

12

## Figures and Tables

**Table:** Key resources table

REAGENT or RESOURCE	SOURCE	IDENTIFIER
**Antibodies**		
human MCTS1	Teleman Lab	N/A
human Tubulin	Sigma Aldrich	Cat# T9026, RRID:AB_477593
human DENR	Teleman Lab	N/A
human GAPDH	Santa Cruz	Cat# sc-47724 HRP, RRID:AB_627678
human Vinculin	Santa Cruz	Cat# sc-73614 HRP, RRID:AB_1131294
human JAK2	Cell Signaling Technology	Cat# 3230, RRID:AB_2128522
human JAK1	Cell Signaling Technology	Cat# 3332, RRID:AB_2128499
human TYK2	Santa Cruz	Cat# sc-5271, RRID:AB_628419
human pSTAT1	BD	Cat# 612133, RRID:AB_399504
Human pSTAT3	Cell Signaling Technology	Cat# 9134S, RRID:AB_331589
Human pSTAT1-PE (pY701)	BD	Cat# 612564, RRID:AB_399855
Human pSTAT4-PE (pY693)	BD	Cat# 562073, RRID:AB_10895804
Human pSTAT3-PE (pY705)	BD	Cat# 612569, RRID:AB_399860
Anti-Human gamma delta TCR – Alexa 647	BioLegend	Cat# 331214, RRID:AB_1089210
Anti-human CD3 – V450	BD Biosciences	Cat# 560365, RRID:AB_1645570
Brilliant Violet 605 anti-human CD56 (NCAM) (Clone 5.1H11)	BioLegend	Cat# 362538, RRID:AB_2565856
BUV563 Mouse Anti-Human CD4 Clone SK3 (also known as Leu3a)	BD Biosciences	Cat# 612912, RRID:AB_2870197
Anti-TCR Vδ1-FITC, human	Miltenyi Biotec	Cat# 130-118-362, RRID:AB_2751495
BUV737 Mouse Anti-Human CD8 Clone SK1	BD Biosciences	Cat# 612754, RRID:AB_2870085
APC/Fire 750 anti-human TCR Vδ2 Antibody	BioLegend	Cat# 331420, RRID:AB_2687326
Brilliant Violet 785 anti-human CD20 [2H7]	BioLegend	Cat# 302356, RRID:AB_2566316
Alexa Fluor 700 anti-human TCR Vα7.2 [3C10]	BioLegend	Cat# 351728, RRID:AB_2566337
MR1-5-OP-RU-tetramer tetramer	NIH tetramer core facility	N/A
Anti-TCR Vβ11-APC, human (clone REA559)	Miltenyi Biotec	Cat# 130-108-733, RRID:AB_2653741
BV480 Mouse Anti-Human Invariant NK T Cell	BD Biosciences	Cat# 746788, RRID:AB_2744044
Anti-T-bet Mouse Monoclonal Antibody (PE/Cy7) [clone: 4B10]	BioLegend	Cat# 644823, RRID:AB_2561760
Brilliant Violet 711 anti-human IFN-γ (Clone 4S.B3)	BioLegend	Cat# 502540, RRID:AB_2563506
Brilliant Violet 510 anti-human TNF-α (Clone MAb11)	BioLegend	Cat# 502950, RRID:AB_2565860
PerCP/Cy5.5 anti-human IL-17A [BL168]	BioLegend	Cat# 512313, RRID:AB_961397
Rorγt Mouse anti Human, PE, Clone: Q21 559	BD Biosciences	Cat# 563081, RRID:AB_2686896
Anti-human CD3 – V450	BD Biosciences	Cat# 560365, RRID:AB_1645570
BUV563 Mouse Anti-Human CD4 Clone SK3 (also known as Leu3a)	BD Biosciences	Cat# 612912, RRID:AB_2870197
BUV737 Mouse Anti-Human CD8 Clone SK1	BD Biosciences	Cat# 612754, RRID:AB_2870085
**Bacterial and virus strains**		
*Mycobacterium bovis* BCG	(Vogt and Nathan, 2011, PMID: 21911939)	N/A
Stellar^™^ cells	Takara	Cat# 636763
**Biological samples**		
Peripheral blood mononuclear cells from indicated individuals	This manuscript	N/A
Plasma from indicated individuals	This manuscript	N/A
**Chemicals, peptides, and recombinant proteins**		
Recombinant human Interleukin-12	R&D Systems	Cat# 219-IL-025
Recombinant human Interleukin-23	R&D Systems	Cat# 1290-IL-010
Recombinant human Interleukin-2	Gibco,	Cat# PHC0023
Recombinant human Interleukin-4	R&D Systems	Cat# 204-IL
Recombinant human M-CSF	R&D Systems	Cat# 216-MC
Recombinant human GM-CSF	R&D Systems	Cat# 215-GM
Recombinant human RANKL	Peprotech	Cat# 310-01
FcBlock	Miltenyi Biotec	Cat# 130-059-901
Phorbol 12-myristate 13-acetate	MedChem Express	Cat# HY-18739
Lymphoprep	StemCell	Cat# 07801
Cycloheximide	Santa-Cruz	Cat# sc-3508
DNase I, RNase-free	Thermo Scientific	EN0521
RNase I	Ambion	AM2294
GlycoBlue	Ambion	AM9515
Acid phenol–chloroform	Ambion	AM9722
Formaldehyde 16%	Thermo Fisher	Cat# 28908
DSP	Thermo Fisher	Cat# A35393
Nonidet P40 Substitute	AppliChem	Cat# A1694
Ionomycin calcium salt	Sigma-Aldrich	Cat# I3909-1ML
Live/Dead Aqua	Thermo Fisher	L34966
Protamine sulfate	Merck	Cat# P3369-10G
Aqua Dead Cell Stain kit	Thermo Fisher Scientific	Cat# L34957
HEK-BlueTM Selection	Invivogen	Cat# hb-sel
QUANTI-Blue Solution	Invivogen	Cat# rep-qbs3
**Critical commercial assays**		
SureSelect Human All Exon V6	Agilent	Cat# 5190-8864
RiboZero TruSeq Stranded Total RNA Library Prep Kit	Illumina	Cat# 20020596
10X Genomics Chromium chip	10X Genomics	NA
Chromium Single Cell 3’ Reagent Kit (v3 Chemistry)	10X Genomics	Cat# CG000183 Rev
Quick-RNA Micro-Prep Kit	Zymo	Cat# R1051
Super-Script II Reverse Transcriptase	Thermo Fisher Scientific	Cat# 18064014
Agilent RNA 6000 Nano Kit	Agilent	Cat# 5067-1511
CD14 MicroBeads	Miltenyi	Cat# 130-050-201
Agilent Small RNA Kit	Agilent	Cat# 5067-1548
SMARTer smRNA-Seq Kit for Illumina	Takara	Cat# 635031
TruSeq Stranded mRNA	Illumina	Cat# 20020594
Maxpar Direct Immune Profiling Assay	Fluidigm	Cat# 201334
TruSeq RNA single indexes	Illumina	Cat# 20020492
Superscript II reverse transcriptase	Thermo Scientific	Cat# 18064022
Qubit dsDNA HS Assay Kit	Invitrogen	Cat# Q32851
Agilent High Sensitivity DNA Kit	Agilent	Cat# 5067-4626
pCR^™^4-T0P0^®^ vector	Invitrogen	Cat# K457502
Dual-Luciferase^®^ Reporter Assay System	Promega	Cat# E1910
Qiagen blood mini kit	Qiagen	Cat# 51104
Lipofectamine 2000	Thermo Fisher	Cat# 11668019
SureSelect Human All Exon 50 Mb kit	Agilent Technologies	Cat# 5190-6213
Big Dye Terminator v3.1 cycle sequencing kit	Applied Biosystems	Cat# 4337455
iPrep PureLink gDNA Blood Kit and iPrep Instruments	Life Technologies, Thermo Fisher Scientific	Cat# 10552894
TaqMan Fast Universal PCR Master Mix (2X), no AmpErase UNG	Thermo Fisher Scientific	Cat# 4352042
Deposited data		
RNA-seq, single-cell RNA-seq, and RIBO-seq	SRA	PRJNA1004232
Experimental models: Cell lines		
HEK293T cells	ATCC	Cat# CRL-11268, RRID:CVCL_1926
HEK-Blue IL-23 sensitive cells	Invivogen	Cat# hkb-il23
HeLa Cells	ATCC	Cat# CRM-CCL-2, RRID:CVCL_0030
THP1 WT Cells	ATCC	ATCC Cat# TIB-202, RRID:CVCL_0006
THP1 MCTS1 KO Cells	This manuscript	NA
Cos-7	ATCC	ATCC Cat# CRL-1651, RRID:CVCL_0224
HT1080	ATCC	ATCC Cat# CCL-121, RRID:CVCL_0317
γ2A- fibrosarcoma cells	Boisson-Dupuis et al. PMID: 30578352	NA
Experimental models: Organisms/strains		
NA	NA	NA
Oligonucleotides		
MCTS1 Exon2+3 Exon trapping Forward	Thermo Fisher Scientific	gatc gaattcCTCATATTCCTCCCCCTAACC
MCTS1 Exon2+3 Exon trapping Reverse	Thermo Fisher Scientific	gatc ggatccCCCATTGTATTCTCAGCACC
Exon Trapping SPL3_MCTS1_Locus Sequencing primer	Thermo Fisher Scientific	aattggttgtagcaggtaata
MCTS1 Site directed mutagenesis of P3 splicing variant forward	Thermo Fisher Scientific	CCTGTCAAAATAGTCCGATGgtctttgtttttgtctgtgt
MCTS1 Site directed mutagenesis of P3 splicing variant forward	Thermo Fisher Scientific	acacagacaaaaacaaagacCATCGGACTATTTTGACAGG
MCTS1 sgRNA1 F	Thermo Fisher Scientific	CACCGGCCTGGGAAGGATACAACAA
MCTS1 sgRNA1 R	Thermo Fisher Scientific	AAACTTGTTGTATCCTTCCCAGGC C
JAK2 Cloning primer F BamH1	Thermo Fisher Scientific	GAGAACCCTGGACCTatgggaatggcctgccttacg
JAK2 Cloning primer R Xho1	Thermo Fisher Scientific	TTTTCTAGGTCTCGAtcatccagccatgttatccc
MCTS1_EXON5+6_P5,7_1-1F	Thermo Fisher Scientific	ggcaacctccaacttttattttg
MCTS1_EXON5+6_P5,7_1-1R	Thermo Fisher Scientific	tgagacaagatcgtgccact
MCTS1_EXON5+6_P5,7_2-1F	Thermo Fisher Scientific	tgtgtaccctgaatcttgactt
MCTS1_EXON5+6_P5,7_2-1R	Thermo Fisher Scientific	gcctcctgagttcaagcaat
MCTS1_EXON5+6_P5,7_3-1F	Thermo Fisher Scientific	ctgctaggtctctgctctca
MCTS1_EXON5+6_P5,7_3-1R	Thermo Fisher Scientific	ggcacatgggttcttttcagt
MCTS1_EXON5+6_P5,7_4-1F	Thermo Fisher Scientific	ctgcacctggtcattactgc
MCTS1_EXON5+6_P5,7_4-1R	Thermo Fisher Scientific	gcccaagtgcattccttctg
MCTS1_EXON5+6_P5,7_5-1F	Thermo Fisher Scientific	ctcaattatttctccccacccc
MCTS1_EXON5+6_P5,7_5-1R	Thermo Fisher Scientific	ttgggaggctgatcacttga
MCTS1_EXON5+6_P5,7_6-1F	Thermo Fisher Scientific	gggacatggacctgctttag
MCTS1_EXON5+6_P5,7_6-1R	Thermo Fisher Scientific	cttcctttgctcatacctgtgt
MCTS1_EXON5+6_P5,7_7-1F	Thermo Fisher Scientific	agactgcctatggagtattagca
MCTS1_EXON5+6_P5,7_7-1R	Thermo Fisher Scientific	agacacactgcctgagagaa
MCTS1_EXON5+6_P5,7_8-1F	Thermo Fisher Scientific	tgggtagtcatgaacatttcca
MCTS1_EXON5+6_P5,7_8-1R	Thermo Fisher Scientific	tgatctcgtggagttgtggt
MCTS1_EXON5+6_P5,7_9-1F	Thermo Fisher Scientific	tgttctcagaactctaccacct
MCTS1_EXON5+6_P5,7_9-1R	Thermo Fisher Scientific	tccagactaagacgtgcataca
MCTS1_EXON5+6_P5,7_10-1F	Thermo Fisher Scientific	tcctcattagcttctgggtgt
MCTS1_EXON5+6_P5,7_10-1R	Thermo Fisher Scientific	ccttccctcttactcatctgga
MCTS1_EXON5+6_P5,7_11-1F	Thermo Fisher Scientific	agtcactttcaagccaggtg
MCTS1_EXON5+6_P5,7_11-1R	Thermo Fisher Scientific	ctcccgggttcatgccac
MCTS1_EXON5+6_P5,7_12-1F	Thermo Fisher Scientific	aagtcatgcaaggagatggc
MCTS1_EXON5+6_P5,7_12-1R	Thermo Fisher Scientific	cctcggtctctcaaagtgct
MCTS1_EXON5+6_P5,7_13-1F	Thermo Fisher Scientific	gtctgtcactcattaaaggcagt
MCTS1_EXON5+6_P5,7_13-1R	Thermo Fisher Scientific	gcacggtcttggctcacc
MCTS1_EXON5+6_P5,7_14-1F	Thermo Fisher Scientific	gtgcatcaaagtcagcccat
MCTS1_EXON5+6_P5,7_14-1R	Thermo Fisher Scientific	gggtgttcttaaagcagggc
MCTS1_EXON5+6_P5,7_15-1F	Thermo Fisher Scientific	tcatgagtggggttagggtt
MCTS1_EXON5+6_P5,7_15-1R	Thermo Fisher Scientific	gtttcgctcttgttgccca
MCTS1_EXON5+6_P5,7_16-1F	Thermo Fisher Scientific	acactctatgcggactcaca
MCTS1_EXON5+6_P5,7_16-1R	Thermo Fisher Scientific	gcacaatctcggctcactg
MCTS1_EXON5+6_P5,7_17-1F	Thermo Fisher Scientific	tggaacttggggaggcttag
MCTS1_EXON5+6_P5,7_17-1R	Thermo Fisher Scientific	tgcatgtgatgatgtatgggg
MCTS1_EXON5+6_P5,7_1-1F	Thermo Fisher Scientific	ggcaacctccaacttttattttg
MCTS1_EXON5+6_P5,7_1-1R	Thermo Fisher Scientific	tgagacaagatcgtgccact
MCTS1 exon2 genomic locus for knockout forward	Thermo Fisher Scientific	GACATTGGTTTTGTGCTGGACAGA
MCTS1 exon2 genomic locus for knockout reverse	Thermo Fisher Scientific	ACCGAGCTTTACACAGACAAAAACA
Cloning-MCTS2 F (BamH1 EcoR1 digest)	Thermo Fisher Scientific	TTGGTACCGAGCTCGGACCTATGTTCAAGAAGTTT
Cloning MCTS2 R( BamH1 EcoR1 digest)	Thermo Fisher Scientific	ATGGATATCTGCAGAATCATTTATATGTCTTCATG
Recombinant DNA		
pCDNA3.1 EV	Thermo Fisher	V79020
pCDNA3.1 MCTS1	This Manuscript	NA
pcDNA3.1 MCTS1 P77AFs	This Manuscript	NA
pCDNA3.1 MCTS1 R72*	This Manuscript	NA
pCDNA3.1 MCTS1 K4-C55	This Manuscript	NA
pCDNA3.1 MCTS1 A133-K181	This Manuscript	NA
pCDNA3.1 MCTS1 A109D	This Manuscript	NA
pCDNA3.1 MCTS1 G24C	This Manuscript	NA
pCDNA3.1 MCTS1 I30M	This Manuscript	NA
pCDNA3.1 MCTS1 E37D	This Manuscript	NA
pCDNA3.1 MCTS1 K46R	This Manuscript	NA
pCDNA3.1 MCTS1 C55S	This Manuscript	NA
pCDNA3.1 MCTS1 R72K	This Manuscript	NA
pCDNA3.1 MCTS1 R83K	This Manuscript	NA
pCDNA3.1 MCTS1 I102V	This Manuscript	NA
pCDNA3.1 MCTS1 S107G	This Manuscript	NA
pCDNA3.1 MCTS1 P119S	This Manuscript	NA
pCDNA3.1 MCTS1 E137K	This Manuscript	NA
pCDNA3.1 MCTS1 Q140P	This Manuscript	NA
pCDNA3.1 MCTS1 A142T	This Manuscript	NA
pCDNA3.1 MCTS1 D154H	This Manuscript	NA
IFNG 5’UTR Reporter	This Manuscript	NA
IL12A-1 5’UTR Reporter	This Manuscript	NA
IL12A-2 5’UTR Reporter	This Manuscript	NA
IL12B-1 5’UTR Reporter	This Manuscript	NA
IL12B-2 5’UTR Reporter	This Manuscript	NA
IL23A-1 5’UTR Reporter	This Manuscript	NA
ISG15 5’UTR Reporter	This Manuscript	NA
IFNGR1-1 5’UTR Reporter	This Manuscript	NA
IFNGR2-1 5’UTR Reporter	This Manuscript	NA
IFNGR2-2 5’UTR Reporter	This Manuscript	NA
IFNGR2-3 5’UTR Reporter	This Manuscript	NA
IL12RB1-1 5’UTR Reporter	This Manuscript	NA
IL12RB1-2 5’UTR Reporter	This Manuscript	NA
IL12RB2-1 5’UTR Reporter	This Manuscript	NA
IL12RB2-2 5’UTR Reporter	This Manuscript	NA
IL12RB2-3 5’UTR Reporter	This Manuscript	NA
IL23R 5’UTR Reporter	This Manuscript	NA
CD40-1 5’UTR Reporter	This Manuscript	NA
CD40-2 5’UTR Reporter	This Manuscript	NA
CD40LG-1 5’UTR Reporter	This Manuscript	NA
CD40LG-2 5’UTR Reporter	This Manuscript	NA
ITGB2-2 5’UTR Reporter	This Manuscript	NA
ITGB2-3 5’UTR Reporter	This Manuscript	NA
PDCD1 5’UTR Reporter	This Manuscript	NA
JAK1-2 5’UTR Reporter	This Manuscript	NA
JAK1-3 5’UTR Reporter	This Manuscript	NA
JAK2-1 5’UTR Reporter	This Manuscript	NA
TYK2-1 5’UTR Reporter	This Manuscript	NA
TYK2-2 5’UTR Reporter	This Manuscript	NA
TYK2-3 5’UTR Reporter	This Manuscript	NA
IRF1-1 5’UTR Reporter	This Manuscript	NA
IRF1-2 5’UTR Reporter	This Manuscript	NA
IRF4 5’UTR Reporter	This Manuscript	NA
IRF8-1 5’UTR Reporter	This Manuscript	NA
STAT1-1 5’UTR Reporter	This Manuscript	NA
STAT1-2 5’UTR Reporter	This Manuscript	NA
STAT1-3 5’UTR Reporter	This Manuscript	NA
ZNFX1-1 5’UTR Reporter	This Manuscript	NA
ZNFX1-2 5’UTR Reporter	This Manuscript	NA
ZNFX1-3 5’UTR Reporter	This Manuscript	NA
REL-1 5’UTR Reporter	This Manuscript	NA
REL-2 5’UTR Reporter	This Manuscript	NA
RORC-1 5’UTR Reporter	This Manuscript	NA
RORC-2 5’UTR Reporter	This Manuscript	NA
CYBB-1 5’UTR Reporter	This Manuscript	NA
IKBKG-1 5’UTR Reporter	This Manuscript	NA
IKBKG-2 5’UTR Reporter	This Manuscript	NA
SPPL2A-1 5’UTR Reporter	This Manuscript	NA
TKB21-1 5’UTR Reporter	This Manuscript	NA
**Software and algorithms**		
R	The R Project for Statistical Computing	https://www.r-project.org
Custom software for sequencing analysis	Aurelio Teleman (github)	https://github.com/aurelioteleman/Teleman-Lab
Uni-form Mani-fold Approximation and Projection (UMAP)	Becht et al. (PMID 30531897)	v.0.3.5
Burrows-Wheeler aligner	Li et al. PMID: 19451168	V0.7.12
GATK	Van der Auwera, G. & O’Connor, B. Genomics in the Cloud: Using Docker, GATK, and WDL in Terra (1st Edition). (O’Reilly Media, 2020).	N/A
VEP (Variant Effect Predictor, Ensemble 37)	Wang et al. PMID: 20601685	N/A
SnpEff-4.1a tools	Cingolani et al. PMID: 22728672	V4.1a
Fastqc	Babraham Bioinformatics	https://www.bioinformatics.babraham.ac.uk/projects/fastqc/
Cutadapt	Martin, M. Cutadapt removes adapter sequences from high-throughput sequencing reads. EMBnet.journal 17, 10–12 (2011).	N/A
Bowtie 2	Langmead & Salzberg PMID: 22388286	Version 2
SAMtools	Li et al. PMID: 19505943	N/A
BEDtools	Quinlan & Hall PMID: 20110278	N/A
BBmap	sourceforge.net/projects/bbmap/	N/A
DESeq2	Love et al., 2014 PMID: 25516281	https://bioconductor.org/packages/release/bioc/html/DESeq2.html
Cell Ranger	10X Genomics	https://support.10xgenomics.com/single-cell-gene-expression/software/pipelines/latest/what-is-cell-ranger
STAR (2.6.1d)	Dobin et al. PMID: 23104886	https://github.com/alexdobin/STAR
Seurat R package	Stuart et al., 2019 PMID: 31178118	https://cran.r-project.org/web/packages/Seurat/index.html
Uniform Manifold Approximation and Projection (UMAP)	Becht et al., 2018 PMID: 30531897	https://cran.r-project.org/web/packages/uwot/index.html
SingleCellExperiment		https://bioconductor.org/packages/release/bioc/html/SingleCellExperiment.html
SingleR		https://bioconductor.org/packages/release/bioc/html/SingleR.html
muscat	Crowell et al. PMID: 33257685	https://bioconductor.org/packages/release/bioc/html/muscat.html))
PLINK	Purcell et al. PMID: 17701901	N/A
logistf		https://cran.r-project.org/web/packages/logistf/index.html
**Other**		
NA		

## Highlights

- X-linked recessive complete human MCTS1 deficiency underlies mycobacterial disease
- A lack of MCTS1 inhibits JAK2 translation by impairing ribosome recycling at two uORFs
- Decreases in JAK2 protein levels impair the responses of NK and T lymphocytes to IL-23
- Innate-like T cells from patients produce low levels of IFN-γ upon IL-23 stimulation
